# Phylogenetic Signals from Nepomorpha (Insecta: Hemiptera: Heteroptera) Mouthparts: Stylets Bundle, Sense Organs, and Labial Segments

**DOI:** 10.1155/2014/237854

**Published:** 2014-04-24

**Authors:** Jolanta Brożek

**Affiliations:** Department of Zoology, Faculty of Biology and Environmental Protection, University of Silesia, Bankowa Street 9, 40-007 Katowice, Poland

## Abstract

The present study is a cladistic analysis of morphological characters focusing on the file of the mandible, the apices of the maxillae, the rupturing device on the maxillae, the internal structures of the mouthparts, and the external morphology of the labial segments as well as the distribution of labial sensilla in true water bugs (Hemiptera: Heteroptera, infraorder Nepomorpha). The study is based on data referring to sixty-two species representing all nepomorphan families (Heteroptera), together with one outgroup species representing the infraorders Gerromorpha (Mesoveliidae). The morphological data matrix consists of forty-eight characters. The present hypothesis supports the monophyly of the Nepomorpha and the monophyly of all families. The new modification in the systematic classification has been proposed: ((Nepidae + Belostomatidae), (Diaprepocoridae + Corixidae + Micronectidae), (Ochteridae + Gelastocoridae), Aphelocheiridae, Potamocoridae, Naucoridae, Notonectidae, and (Pleidae + Helotrephidae)).

## 1. Introduction

The classification system of true bugs Heteroptera recognizes seven major taxonomic groups, usually referred to as infraorders (Enicocephalomorpha, Dipsocoromorpha, Gerromorpha, Nepomorpha, Leptopodomorpha, Pentatomomorpha, and Cimicomorpha) [1–3], or eight infraorders after the addition the Aradimorpha sensu Sweet [4, 5].

As far as the approach to cladistic relationships among infraorders of the Heteroptera is concerned, several various hypotheses have been proposed with respect to the systematic position of the Nepomorpha as well as other infraorders (generally without considering the Aradimorpha) and discussed in phylogenetic studies.

According to Schuh [6] the Enicocephalomorpha (first branch) is a basal, sister group to all remaining infraorders of the Heteroptera. In the following branches the Dipsocoromorpha, Gerromorpha, Nepomorpha, Leptopodomorpha, Cimicomorpha, and Pentatomomorpha have been placed. In this arrangement the Gerromorpha is the sister group to the Nepomorpha.

Similarly, on the basis of morphological evidence, Štys [7, 8] placed the Enicocephalomorpha in the most basal clade of the Heteroptera. In Zrzavy's [9] system of relationships it was indicated that the Enicocephalomorpha was the sister group of Dipsocoromorpha + Gerromorpha and together formed a basal heteropteran clade in relation to the unresolved relationships (polytomy) among the Nepomorpha, Leptopodomorpha, and Cimicomorpha + Pentatomomorpha.

Furthermore, on the basis of morphological characters, Mahner [10] proposed a hypothesis that the Nepomorpha (Cryptocerata) should be placed as the basal, sister taxon to the remaining Heteroptera which also coincided with the hypothesis of Shcherbakov and Popov [11], based on fossil morphological evidence, although in both cases unresolved relationships were notated among the remaining infraorders.

Wheeler et al. [12] generally reached a substantial congruence between the molecular data and most of the morphological data used by Schuh [6] in the system of classification of heteropteran infraorders, even though a distinct result seemed to be the establishing of the sister clade Nepomorpha + Leptopodomorpha (as in Figure 6 in [12]). In the classifications of infraorders based on characters of male genitalia Yang [13] pointed out that the Enicocephalomorpha was the sister group to the remaining Heteroptera; however, in the arrangement such as the Enicocephalomorpha + (Leptopodomorpha + Cimicomorpha + Pentatomomorpha) + (Dipsocoromorpha + (Nepomorpha + Gerromorpha)), the above mentioned groups were presented as three unresolved branches. Recently, in the infraordinal relationships based on whole sequences of 18S rDNA whose alignment was modified by the secondary structure of rRNA, Xie et al. [14] obtained results featuring single branches for the Enicocephalomorpha, Nepomorpha, Leptopodomorpha, and two clades: Gerromorpha + Dipsocoromorpha and Cimicomorpha + Pentatomomorpha. Besides, using 64 morphological characters and DNA sequence data from the mitochondrial genes encoding COI+II and 16S rRNA and the nuclear gene encoding 28S rRNA, Damgaard [15] established the relationship of (Enicocephalomorpha + (Dipsocoromorpha + (Gerromorpha + Nepomorpha))). On the basis of multiple genes in many species of the heteropteran infraorders, Li et al. [16] revealed that the Nepomorpha was the most basal group.

Several different systematic position of the Nepomorpha within the Heteroptera have been indicated and essentially, only in three studies, the Gerromorpha has been estimated as the outgroup (sister clade) to the nepomorphans [6, 12, 15].

In historical views, various hypotheses have been proposed with respect to the relationships within the taxa of the Nepomorpha. In the first evaluations of the relationships among the true water bugs [20], the Corixidae were considered to be primitive and treated as a sister group of the remaining families. Later, China [21] proposed a scheme of the relationships among nepomorphan families in which the Ochteridae were treated as relatively the most primitive group based on the possession of ocelli and a respiratory system typical of terrestrial bugs. Comparative studies of the mouthparts [19, 22, 23], the egg structures, and other characters of embryology of the Heteroptera [24] supported the hypothesis that had been proposed by China [21]. Furthermore, according to several authors such as Popov [25], Rieger [26], Mahner [10], and Hebsgaard et al. [27], the Belostomatidae and Nepidae (Nepoidea) were introduced at the basal position and estimated as a sister group of the remaining nepomorphan families. However, their scenario of relationships was essentially different with respect to other nepomorphan families. Popov [25], Mahner [10], and Hebsgaard et al. [27] placed the Corixidae (Corixoidea) as the second group (second branch) in the arrangement of relationships system. In Rieger's [26] system the clade Ochteridae + Gelastocoridae (Ochteroidea) was shown in the second branch, while the Corixidae were presented as the third branch. A similar concept of the relationships among the Potamocoridae, Naucoridae, and Aphelocheiridae could be seen in systems developed by Popov [25] and Mahner [10]. Rieger [26] indicated the clade Naucoridae + Potamocoridae; however, Hebsgaard et al. [27] indicated the clade Aphelocheiridae + Potamocoridae and ranked it as a new superfamily, Aphelocheiroidea; furthermore, they placed the Ochteridae and Gelastocoridae (Ochteroidea) in a new position, that is, as a branch under the Naucoridae (Naucoroidea). Popov [25], Rieger [26], Mahner [10], and Hebsgaard et al. [27] generally agreed in the classification and relationships of the Notonectidae, Pleidae, and Helotrephidae. The new relationships of some families of the Nepomorpha postulated by Hua et al. [28] are interesting due to the specific location of the Pleidae. According to these authors, the Pleidae derive from the Nepomorpha as a new heteropteran infraorder; the Plemorpha and the monophyletic infraorder Nepomorpha consist of five superfamilies with the following relationships: (Corixoidea + ((Naucoroidea + Notonectoidea) (Ochteroidea + Nepoidea)). Nevertheless, not all families which were recognized within the Nepomorpha in the study by Hua et al. [28] have been analysed so far. Moreover, other relationships of super(families) of the Nepomorpha based on four Hox genes have been indicated by Li et al. [29]. According to their study, the most basal lineage is the Ochteroidea, whereas the Notonectoidea include only the Notonectidae and form a new sister relationships with (Pleoidea + Naucoroidea) and (Nepoidea + Corixoidea).

Members of the true bug group of the Nepomorpha have attracted the attention of researchers by displaying a variety of body structure forms and lifestyles. All of these subjects have been widely discussed in literature. However, the number of papers dealing with nepomorphan (sub)family-level relationships still remains relatively small and the phylogenetic affinities of some family groups still require clarification.

The water bugs classified as the Nepomorpha include about 2000 species worldwide [30], and as for the composition of this infraorder, there are 13 families. In the classification of Štys and Jansson [31] 11 families of the Nepomorpha were distinguished. Two subfamilies (Diaprepocorinae and Micronectinae) that belonged to the Corixidae were elevated to the rank family level of the Micronectidae and Diaprepocoridae by Nieser [32] and have been accepted at that position by other researchers [33–37].

In order to meet the requirements of the many ways of life adopted by members of the Nepomorpha, the morphology of the species in various families displays a great variety of modifications [25, 38]. The representatives of most families live in water (aquatic bugs), except for the gelastocorids and ochterids, which occupy habitats at the water's edge [3, 25, 30, 39–42], like the Saldidae of the Leptopodomorpha. Those that remain submerged include fast swimmers inhabiting the open water, including the corixids and notonectids [38, 43–45] and slow-moving benthic species that breathe through long respiratory siphons, such as many nepids and belostomatids [25]. Morphologically, the group is characterized by the shortness of the antenna that is typically concealed, either partly or entirely, by the eyes [25, 30, 46] and families of most species can be identified immediately on the basis of size and general body shape. The general morphology is usually similar throughout the larval stages, and the family affiliation of the first instar larvae can immediately be recognized after only a cursory examination in almost all cases [38, 47]. However, the general morphology of corixids differs in several ways from that of other groups of true bugs [25, 48].

Some of the Nepomorpha species are mostly predators whereas most corixids are plant feeding; however, there are several species which prefer feeding on animals or a mixed type of nutrition [43–45, 49–51].

So far, a significant range of various studies have been conducted with regard to the nepomorphans. The most comprehensive papers on the subject have been written by China [21] on general biology and morphology of water bugs; Popham [52] on the respiration of aquatic bugs; Cobben [19, 24] on embryology and eggs, male genitalia, and mouthpart structures of the Heteroptera; Parsons [22, 23, 48] on triturating device, salivary pump, thorax, and labial skeleton; Popov [25] on general morphology and fossils study; Rieger [26] on the structures of the head and prothorax of* Ochterus*; Cassis and Silveira [53] on morphology and interrelationships in the Gelastocoridae (Nerthrinae). Also, the principal work of Mahner [10] included a number of comprehensive useful data on morphology within the Nepomorpha and their phylogeny. The first studies combining the morphological and genetic data in the Nepomorpha were conducted by Hebsgaard et al. [27] and by Hua et al. [28], who studied phylogenetic relationships based on the genomes. Generally, many other studies focusing on various fields of morphology and biology of the nepomorphans have been conducted by researchers.

Comprehensive studies of characters of the labium in the nepomorphans and in several individual species of the Corixoidea were conducted by Parsons [22, 48] and previously by Griffith [54], Bentwitz [55], and Puchkova [56]. The papers of the above mentioned authors generally presented researches focusing strictly on the labium.

The studies by Brożek ([57–59], 2014 in press) have also provided a number of useful new observations on the morphology of maxillae and mandibles, labial sensilla and labial segments within the Nepomorpha, which can be considered from the phylogenetical perspective of this infraorder.

The results achieved in these studies allowed establishing many new features in comparison to previous researches (the present compilation of data is meant to provide a summarizing description of characters). The use of the available data in the present analysis of the characters is justified by the fact that in the last combined phylogenetic analysis (using morphological and molecular data) conducted by Hebsgaard et al. [27] only four characters of the labium were incorporated. Moreover, the labial sensilla as well as the maxillary and mandibular structures were not previously estimated in the cladistic analysis of the Nepomorpha. The evaluation of all these characters was conducted only on the basal ground plan of assumptions ([57–59]). For this reason, the recent new descriptions of new characters of the mouthparts have provided an opportunity for reassessing the phylogenetic relationships within nepomorphan groups through conducting new analyses based on the available new data.

In doing so, an opportunity has been taken to reexamine the relationships within the Nepomorpha and to make a comparison between the concept presented by Hebsgaard et al. [27], based on relationships achieved from the morphological data and also from the final combined data (morphological and genetic) and the currently available new strict consensus on the phylogenetical tree. The principal approach of the present cladistic analyses with respect to the concept of Hebsgaard et al. [27] has been possible due to the use of the same methodology in the analyses focusing on a great number of the same species or genera. Furthermore, in the studies of the Nepomorpha conducted by Hua et al. [28] as well as Li et al. [29] different relationships of super(families) were presented based on various molecular data using a cladistic analysis, so the comparison of their results with the present morphological data in this area is also possible. In addition, the present detailed study of these characters in the Nepomorpha also provides an opportunity for comparison with other morphological hypotheses regarding the phylogenetic relationships, proposed by China [21], Popov [25], Rieger [26], and Mahner [10], even though their analyses were not algorithmic analyses.

The main goal of this paper is to clarify the significance of the characters of mouthparts structures (labial segments and sensilla, external and internal structures of maxillae and mandibles) in the relationships of the nepomorphan families based on cladistic analyses.

## 2. Material and Methods

### 2.1. Taxa Sampled

The species listed in Table 1 and used for the purpose of the study came from the collections of the Natural History Museum in Vienna, Zoological Museum of the State Moscow University, and the Paleontological Institute of the Russian Academy of Sciences in Moscow. The new characters presented in this paper have been described based on SEM images of the mouthpart structures. The SEM photographs were taken with a Hitachi scanning electron microscope.

### 2.2. Range of Characters

A preliminary estimation of the characters of the maxillae, mandible structure, and labial sensilla with respect to their phylogenetic value based on the ground plan was compared with the basic model within the group (i.e., the basal taxa of the Nepidae and Belostomatidae) and with the more diverse forms of these structures in more evolutionarily advanced groups (i.e., Ochteridae, Gelastocoridae, Aphelocheiridae, Naucoridae, Pleidae, Helotrephidae, Notonectidae, Diaprepocoridae, Corixidae, and Micronectidae). All these data were taken from papers authored by Brożek [57, 58]. The construction of the labial segments of most nepomorphans was presented by Brożek [59]; moreover, details of morphological characters of the labium in the Corixoidea (Nepomorpha) were also investigated by Brożek (2014 in press). Presently, the total of all characters proposed by Brożek ([57–59], 2014 in press) provides an improved characteristics of these features which can be combined for the purpose of a more precise coding (Tables 2 and 3) with respect to the outgroup and analyzed from the phylogenetic perspective.

### 2.3. Outgroup Selection

In the present study, the outgroup of the Gerromorpha was accepted with respect to the Nepomorpha according to the hypothesis proposed by Wheeler et al. [12]. Different variants of phylogenetic relationships among infraorders of the Heteroptera have been established as mentioned in the Introduction. Additionally, the nepomorphan characters are polarized with respect to the Mesoveliidae, because this family is the most plesiomorphic one within the Gerromorpha [17]. The presently selected species of* Mesovelia* is congruent with the outgroup used in the study by Hebsgaard et al. [27]. Choosing the same outgroup as in the study by Hebsgaard et al. [27] provides a chance for the comparison of morphological characters of mouthparts which is methodologically correct, that is, by identical direction of polarization in the analysis.

### 2.4. Type of Coding

**Figure 1:**
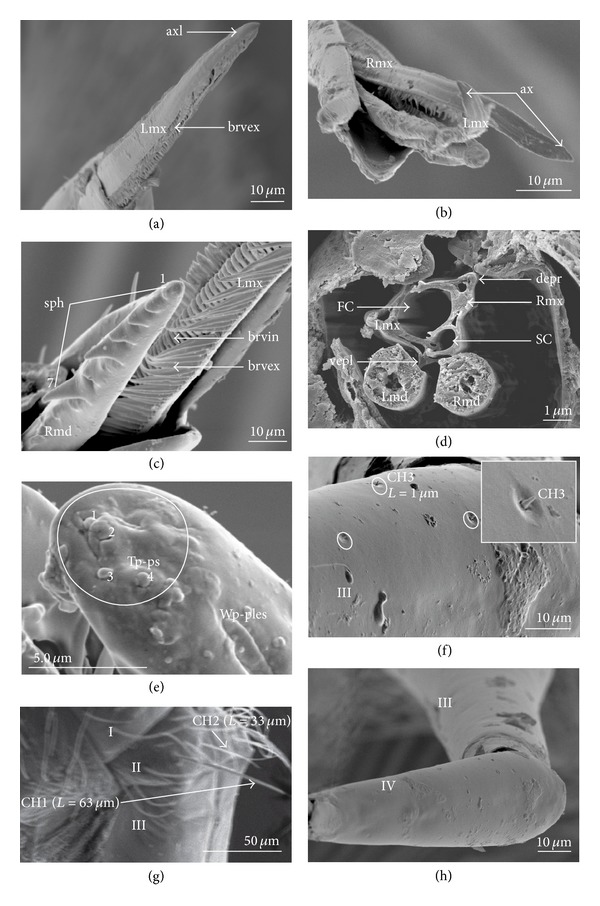
A set of characters for the maxillae, mandibles, and labial sensilla of* Mesovelia* (Gerromorpha). (a) The shape of the apex of maxillae, the left apex (axl) is visible, exposed, the ventral rupturing device (brvex) is visible. (b) The straight and narrow apice (ax) of both maxillae (Rmx, Lmx). (c) Right mandibular file (Rmd) consists of seven short spines (sph); the file is of medium length, two rows of spines in the rupturing device: internal (brvin) and external (brvex). (d) Cross-section of maxillae (Rmx and Lmx) and mandibles (Rmd and Lmd). (e) The labial tip with peg, uniporous sensilla (Tp-ps) and subapically placed elongated, multiporous plate sensillum (Wp-ples). (f) An arrangement of the mechanosensilla, several short chaetica sensilla (CH3) on the third labial segment. (g) Numerous chaetica sensilla, long (CH1) and of medium length (CH2) placed on the first and second segment, dorsally. (h) The sensilla are small and a few are present on the third and fourth segments, FC: food canal; SC: salivary canal; depr: dorsal external process, right; vepl: ventral external process, left.

**Figure 2:**
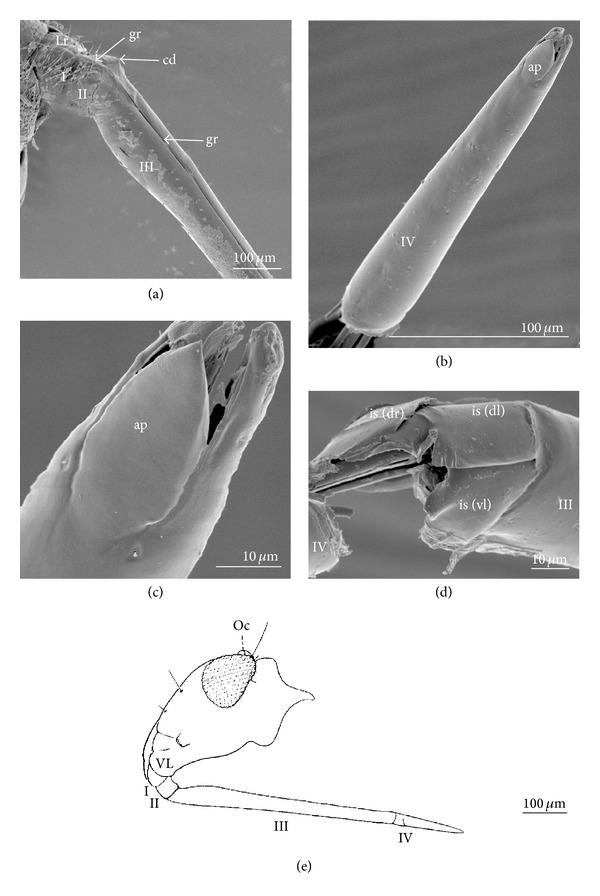
Characters of the labial segments of the* Mesovelia* (Gerromorpha). (a) Shapes of segments: first (I) is ring shaped, well visible from the dorsal side, the second (II) is similar to the first but slightly shorter, and the third (III) is tubular and very long. (b) The fourth segment is tubular, ventral view. (c) The shape of the ventral apical plate (lobe shaped). (d) Shapes of the intercalary sclerites (three large sclerites are visible: dorsal right (is (dr), left (is (dl), and ventral left (is (vl)). (e) The complete view of the labial segment of* Mesovelia mulsanti* (drawing from Andersen [17]).

**Figure 3:**
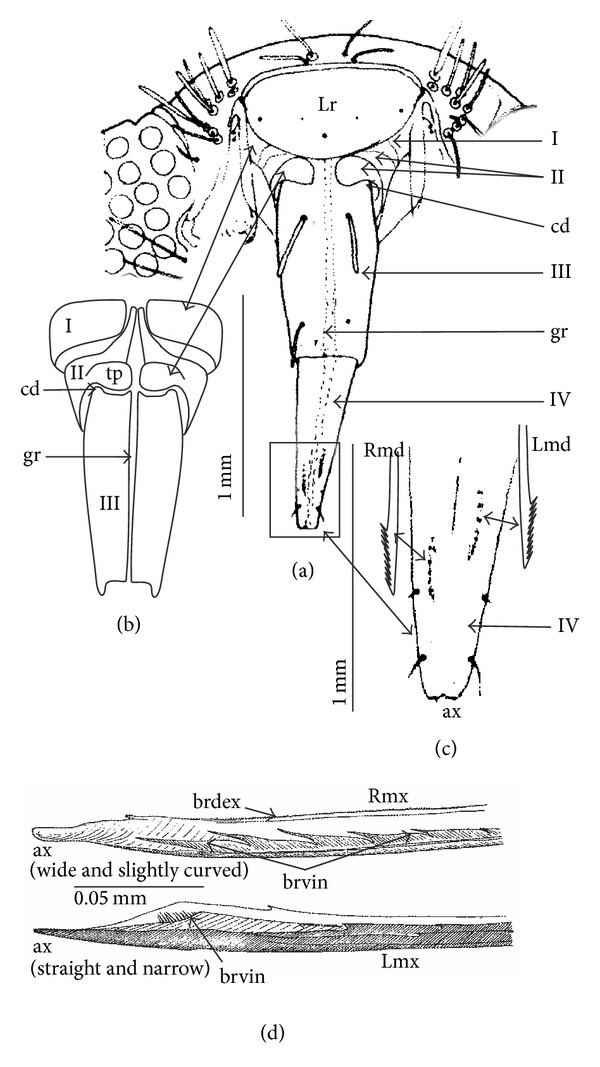
Morphological characters of the labium, maxillary, and mandibular stylets of the Potamocoridae. (a) The labium of* Potamocoris nieseri* according to von Doesburg [18]. (b) Shapes of the first (I) and second (II) segments of the labium of* Potamocoris nieseri* are presented as presumable shapes deduced presently by Brożek. (c) Magnification of the IV segment (black dots inside the segment point out the presence of mandibles). The drawings of mandibles (Rmd and Lmd) showing putative shapes of the mandibular file (a precise description based on the original drawing by von Doesburg [18] was impossible). (d) The shape of the right and left maxillae (Rmx and Lmx) of* Potamocoris sp*. shown on the basis of a drawing taken from Cobben [19]. The right apex (ax) is wider than the left one; the right apex is slightly curved while the left one is straight and narrow. The inner system of spines on the maxillae edges from the reduced and hidden rupturing device (brdex and brvin). On the right maxilla seven short spines are present on the internal ventral side (brvin). On the dorsal side the spines are strongly reduced (brdex). On the left maxilla, internally, one tuft of short spines (brvin) can be observed. I: first segment, II: second segment, III: third segment, IV: fourth segment, ax: apex of maxillae, cd: dorsal condyle (the articulation between the second and third segments on the dorsal surface), gr: labial groove, tp: triangular plate of the second segment, Lr: labrum, Rmd: right maxilla, Lmx: left maxilla, Rmd: right mandible, Lmd: left mandible.

Several characters of the outgroup used for the purpose of this paper originated from the description of the elements of the rostrum by Andersen [17] and Cobben [19] while others were based on the materials prepared presently (description and Figures 1(a)–3(d)) by Brożek. Characters and states selected as being of interest are marked as (Kn (state number K0–K47)). All of them are presented in Tables 2 and 3 for the Nepomorpha and additionally have been illustrated with their different states in Figures 1(a)–3(d) for the outgroup and the Potamocoridae. The analysis included 63 ingroup taxa and one outgroup taxon. A total of 48 characters were scored; 23 of these were binary and 25 were multistate. The morphological characters for all taxa were coded from the examination of specimens by present author, except for the Potamocoridae: their characters were based on the descriptions of previous other authors. Characters (0–47) and their hypothesized states are shown in Table 2. Character states were written into a standard character by taxon character state matrix (Table 3) with unknown characters coded as a question mark (?).

The studied species were coded as having individual characters (Table 3) to provide a more accurate reflection of the observed morphology, rather than trying to achieve uniformity of coding within the (sub)families. The characters used for analysis were based exclusively on adult structures.

The morphological characters presented in Tables 2 and 3 were shown according to the ground plan characters and the outgroup was shown as in the previous studies by Brożek [57–59]. In the present cladistic analysis all these characters were regarded as nonadditive and equally weighted in order to avoid regarding them in an* a priori* manner and to conduct estimation through algorithms adopted by the cladistic software. Additionally, such an estimation was necessary due to the presence of characters coded as unknown (?) in the Potamocoridae.

### 2.5. Programs Used for Cladistic Analysis

Morphological data (Table 3) were analysed using the parsimony programs NONA [60] and Winclada (BETA) ver. 0.9.9 [61] with equal weight characters and heuristic search with TBR transformation option. However, Goloboff [62, 63] presented convincing justification for using implied weights in cladistic analysis and his method has since been widely used, with some authors preferring it to equal weights.

The nonhomoplasies and homoplasies on cladograms were searched using unambiguous and slow optimization in order to evaluate how the character data on the cladogram changed. Winclada, in particular, apply itself to investigating synapomorphies supporting nodes as it allowed for the mapping of all characters and states simultaneously. Additionally, characters were also analysed using the heuristic search option of PAUP*4.0 [64]. All characters were used as nonordered, of equal weight with ACCTRAN transformation option, and character polarities were determined in the context of the phylogenetic analysis. The topology of trees and the arrangement of terminal taxa as well as a length, consistency, and retention index obtained in PAUP and NONA program were similar. NONA [60] and PIWE [65] were also used for the calculation of Bremer support values (decay index) for branches [66]. Runs were conducted using the following commands: Mult*10; Max*; and subsequently HOLD 1000; SUB 1; FIND*; HOLD 2000; SUB 3; FIND*; HOLD 4000; SUB 5; FIND*; HOLD 5000; SUB 15; FIND*; BSUPPORT. Bremer support values, shown in Figure 7, were calculated as measures of branch support up to 15 steps away from the most parsimonious solution. These values were also checked in the TNT program [67].

Bootstrap support implemented in Winclada [68] of 1000 resampling replicates was used to study the level of character support in the dataset for hypothesized clades. Using new TNT technology methods for searching did not result in shorter trees.

### 2.6. Explanation and Documentation of Morphological Characters of the Ingroups of the Nepomorpha and the Outgroup (Gerromorpha: Mesoveliidae:* Mesovelia furcata*)

#### 2.6.1. The Outgroup

Species of Mesoveliidae as well as other representatives of gerromorphan taxa are characterized by their highly serrated maxillae and sharp barbs of the mandibles [19]. Presently, the original photographical documentation of* Mesovelia *(Figures 1(a)–1(h)) indicated that mandibles were evenly serrated apically and equipped with seven short spines (Figure 1(a)). On the basis of such appearance of the mandibular file they were included in the categories of evenly serrated (short spines) and medium length (K0 (0)). The observation of maxillary stylets (Figures 1(b) and 1(c)) showed that the apices were symmetrical (both apices straight and slightly narrow and flat (K1 (0)). The maxillary spines were stiff, long, and forming regular and dense external (brdex and brvex) and internal rows (brvin) along the edges of the maxillae, that is, exposed a rupturing device (Figure 1(a)) (K2 (0)).

In the cross-section (Figure 1(d)), the locked maxillae (Rmx and Lmx) appeared to be pentagonal in shape with the dorsal side distinctly tapered and wider than the ventral side. On the dorsal and ventral sides both had one pair of protuberant external lobe processes (depr, depl, vepr, and vepl) (K3 (0)). The mandibles (Rmd and Lmd) (K4 (0)) were placed on the lateral suboval walls of the maxillae between the dorsal and ventral lobes.

In* Mesovelia* there is substantial variation in the set of labial sensilla in comparison to the representatives of the Nepomorpha. Several short chaetica sensilla (CH3) (mechanosensilla) were found on the dorsal and ventral side of the III and IV segments (Figures 1(e) and 1(f)) (K5 (0)), whereas slightly longer chaetica sensilla (CH2) (K6 (0)) and long chaetica sensilla (CH1) (K7 (0)) were observed on the I and II segments (Figure 1(g)). Essentially, only one pair of the proprioceptive hairs (mechanosensilla) was situated on the ventral and dorsal sides of the II segment (K8 (0)) and one pair on the ventral side (K9 (0)). In* Mesovelia*, the characters mentioned in Table 2 from K10 to K22, K24, and K26 (0) were estimated as absent characters. Near the labial tip on the dorsal and ventral sides one pair of trichoid sensilla (TRS) was found (probably bimodal sensilla: mechanoreceptors and gustatory) (K23 (0) and K25 (0)). The characteristic type of sensilla was a plate-like, elongated sensillum (Wp-ples) (Figure 1(h)) present in the Mesoveliidae (K27 (0)) and the Hebridae, while in the Nepomorpha it was absent [57]. In* Mesovelia* (Figure 1(h)) the peg-in-pit sensilla (poreless coeloconic sensilla) were not identified on the labial tip (K28 (0)); however, four peg sensilla (contact-chemoreceptive sensilla, mechano- and chemoreceptors) were observed centrally on the labial tip (K29 (0) and K30 (0)). These sensilla were inserted in the socket on the smooth surface of the tip (K31 (0)). In this species the sensilla on the labial segments were essentially less numerous and not very distinguished as well as unevenly arranged (K32 (0)). Generally, all these sensilla were classified on the basis of characters distinguished in many publications referring to this subject [58, 69–72].

The labium in* Mesovelia* showed a substantial similarity in structure to the representatives of the Nepomorpha (except for the Corixoidea). The labial apex on the ventral side was equipped with one oval plate (ap) (Figure 2(a)) (K33 (0)), which was similar to some species of the Nepomorpha. The Mesoveliidae (and Hebridae) appeared to be the only group with four large intercalary sclerites (is-dr, is-dl, is-vl, and is-vr (is-vr is invisible only in Figure 2(b)). These sclerites were situated on the distal edge of the third segment, and they surrounded the dorsal and ventral sides of the labium (K34 (0)). The edges of the dorsal surface of the labium were not in contact medially and the stylet groove was open (K35 (0)). The first labial segment was short ventrally and longer dorsally, generally ring shaped (K36 (0)) (Figure 2(c)). The dorsal surface of this segment was covered by the labrum. The second segment on the dorsal side was smooth (i.e., in that part the segment was not divided) (K37 (0)) (Figure 2(c)) and the dorsal edges of the segment were in contact, so that the stylet groove was closed (K38 (0)). Also the lateral surface of the segment was uniform (without no incision) (K39 (0)). The stylet groove of the two basal segments of the labium was covered by an epipharyngeal projection. The second segment was usually the smallest of the four labial segments (Figure 2(c)) (K40 (0)). The third labial segment was by far the longest (K41 (0)). Typically, it was swollen proximally and tapered distally. The fourth segment was distally shorter than the preceding segment and tapering towards the pointed apex (K42 (0)). Ventrally, the distal edge of the first segment was hidden and the midventral condyle was estimated as lack of data (K43 (?)); on the third segment the midventral condyle was putatively present (K44 (0)). Evidently, the condyle was not observed on the proximal edge of the fourth segment (K45 (0)) (Figure 2(d)). The second segment was connected with the third segment dorsally by a wider band of membrane (K46 (0)), dorsal articulation (cd)) (Figure 2(c)). The labium was four segmented and tubular shaped (K47 (0)) (Figure 2(e)).

#### 2.6.2. Nepomorpha: Potamocoridae

The set of characters required for the present analysis was selected on the basis of data from literature.

The Potamocoridae are basically a group which has been scarcely investigated with respect to their morphology. Several studies focused only on the general morphology of the body [3, 18, 19, 73–76]; however anatomical details of particular elements of the body parts are not known. In the studies of the Nepomorpha conducted by Brożek [57–59] the Potamocoridae were not analyzed, as the material of those families was unavailable. Due to this, in the present study only several characters were analyzed which had been described previously by several authors. In the Potamocoridae most of characters (K) mentioned in the Table 2 were coded as unknown (lack of data).

According to Cobben [19] maxillary stylets of* Potamocoris sp*. (Figure 13(B), pp 36-37) are structurally entirely different from the typical naucorid stylets. On the basis of the review of many maxillary stylets of nepomorphan taxa [57] it is possible to compare the maxillary stylets of* Potamocoris sp*. with other nepomorphan species. On the basis of their appearance, maxillary stylets of* Potamocoris sp*. (Figure 3(a)) were classified as stylets with asymmetrical apices (the right one (Rmx) was straight and narrow; the left one (Lmx) was wide and curved) (K2 (4)) like the Aphelocheiridae and Cheirochelinae (*Coptocatus oblongulus*,* Coptocatus kinabalu*,* Cheirochela feana,* and* Gestroiella limnocoroides*). On the internal edges of the maxillae in* Potamocoris sp*. there were several short spines (seven on the right maxilla (Rmx) (brdex and brvin) and one tuft with minor spines (brvin) on the left maxilla (Lmx)). When the maxillae were locked the spines were externally invisible. Such a system of spines was evaluated as the rupturing device almost reduced and hidden (K3 (9)), like the Corixoidea, Pleidae, and Helotrephidae [57]. The coded characters from K4 (?) to K34 (?) were treated mainly as a lack of data and referred to labial sensilla.

According to van Doesburg [18] the labium of* Potamocoris nieseri* was broad at the base, tapering to the tip of its third segment. The last segment was slightly shorter than the second one. On the basis of the drawing by van Doesburg [18] (Figure 2(a) (*Potamocoris nieseri*), pp. 22) it was possible to estimate that there were no intercalary sclerites (K35 (8)) (Figure 3(b)). A similar conclusion was drawn by Cobben [19]. Even though the drawing was based on the picture from the light microscope, certain structures of the labium could be recognized and compared to the SEM images of the labium of other nepomorphans. According to my experience, the drawing of* Potamocoris nieseri* showed the fourth segment of the labium and its appearance was similar to the labium of naucorids and pleids.

I took the liberty of describing the drawing made by van Doesburg [18] according to categories, which were used by Brożek [59], and to introduce these characters to the present analysis. The first segment (I) and partly the second (II) segment were covered by the triangular labrum (Lr). There was no certainty as to the type of the stylet groove of the first segment, so that the K36 (?) was estimated as a lack of data. The first segment was rather narrow (K37 (1)); the lateral sides were visible and reaching to the base of the labrum (Figures 3(b) and 3(c)). In Figure 3(b), the laterally and dorsally visible elements belonging to the second segment (II) corresponded to the elements (tp and cp) putatively marked in Figure 3(c). The dorsal surface of the second segment in this species could be divided into a triangular, flat plate (tp) and a second plate (cp) (K38 (1)). They were placed symmetrically on the left and right side of the stylet groove. In the second segment the stylet groove was usually open up to the half-length of the segment (K39 (2)) (Figure 3(c)). For the* Potamocoris* it was assumed that the lateral surface of the second segment was smooth (K40 (0)) as in most nepomorphans. The first and second segments were short (K41 (0). The third segment (III) (mentioned as the second one by van Doesburg [18]) was long (K42 (1)) in comparison to the first and second segment, and the fourth one was shorter than the third (K43 (0)). The midventral condyle (K44 (?), K45 (?), and K46 (?)), either present or absent in various nepomorphans, was estimated as a lack of data for* Potamocoris nieseri*. Dorsally, the third and second segment had two points of articulation (band shaped (K47(0)). On the basis of Figure 3(b) it could be suggested that the labium was four segmented and tubular shaped (K48 (0).

### 2.7. Number of Codes and State Definitions of Characters

#### 2.7.1. Characters (K0–4): The Shapes of Maxillae and Mandibular Stylets of the Nepomorpha according to Brożek [57] (Table 2)

General stylet structures were used in a prior cladistic analysis of relationships within the Heteroptera by Cobben [19]. Presently, the condition of stylets found in the Nepomorpha indicated a substantial variation in stylet structure within the group. Ten morphologically distinct types of files (K0) were identified on the mandibular tip in individual species, as well as eight distinct types of maxillary endings (K1) in individual species and ten distinct types of rupturing devices (K2) of the maxillae. The features of the internal maxillary (K3) and mandibular (K4) structures shared a common connection model, differing only by virtue of specific appendages in different subfamilies.

#### 2.7.2. Characters (K5–32): Labial Sensilla Types and Distribution Patterns of Sensilla in the Nepomorpha according to Brożek [58] (Table 2)

Twenty-one morphologically distinct types of the mechanosensilla as well as two types of the trichoid sensilla (contact-chemoreceptive sensilla) were identified on all labial segments in representatives of the subfamilies. The chaetica sensilla (CH3, CH2, and CH1) were present in various layouts on the segments (K5, K6, and K7). The proprioceptive sensilla were positioned on the dorsal side (K8) (either one pair or more pairs) and on the ventral side (K9) (one pair) on the second segment of the labium. Several variously shaped mechanosensilla were specific for individual species (K10–K22). Variously shaped trichoid sensilla are placed on the IV segment (K23, K24, and K25) and on the III segment (K26). Near to the labial tip, subapically, the elongated plate sensillum was present in representatives of the outgroup (K27); however, it was absent in the Nepomorpha. On the labial tip of the nepomorphans, three morphologically distinct types of chemosensilla were identified: one type of the peg-in-pit sensilla (K28) and two types of papillae sensilla (K29), as well as various types of their distribution. In addition, these sensilla were present in various numbers, from a few to a dozen (K30). The sensilla were inserted in the labial tip, either smooth or folded (K31). The mechanosensilla were present and placed in groups or rows distributed along the labium near the labial groove on the dorsal side; the sensilla were also unevenly scattered over the ventral surface of that segments (K32).

#### 2.7.3. Characters (K33–47): Shape of the Labial Segments of the Nepomorpha according to Brożek [59] (Table 2) 

Within the thirteen families, six morphologically distinct forms of the apical plate (K33) of the labium and several intercalary sclerites (K34) were identified. Although in most investigated taxa of the nepomorphans subsequent segments of the labium (I, II, III, and IV) were shaped similarly, individual characters in some (sub)families differed (K35–42). The presence of the midventral condyle on the distal edge of the first segment (K43) and the third segment (K44) was observed, but not in all species. A new position of the midventral condyle on the proximal edge of the fourth labial segment (K45) was distinguished in several groups. Additionally, three types of articulation (K46) on the dorsal side between the third and second segments were interpreted as the new characters in relation to previous studies of this area.

The labium showed a substantial variation in the structure and segmental development between the Corixoidea and the remaining nepomorphans. The Corixoidea appeared to be the only group in which the first and second segment were completely lost on the dorsal side. Generally, the labium is triangular-shaped and short (K47)); however there had been evidence that the third and fourth segments were conspicuously present ([77]; Brożek, 2014 in press).

## 3. Results

### 3.1. Morphological Characters Mapped on the Parsimonious Tree

Character analysis (complete data matrix presented in Tables 2 and 3).

**Figure 4:**
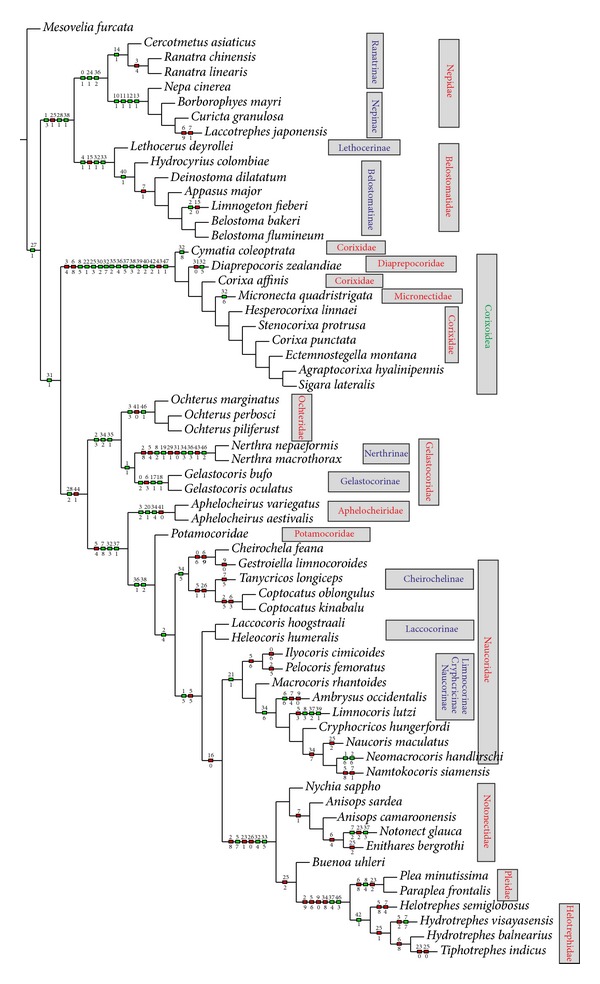
The most parsimonious tree resulting from the heuristic search with characters treated as unordered and equally weighted (tree length = 198, consistency index = 71, retention index = 92). A small green box indicates nonhomoplasy (synapomorphies and autapomorphies); a red small box indicates homoplasy. The number above the branch line refers to the number of a character; the number below the line of the branch refers to number of the state of a character. The unambiguous option is used.

**Figure 5:**
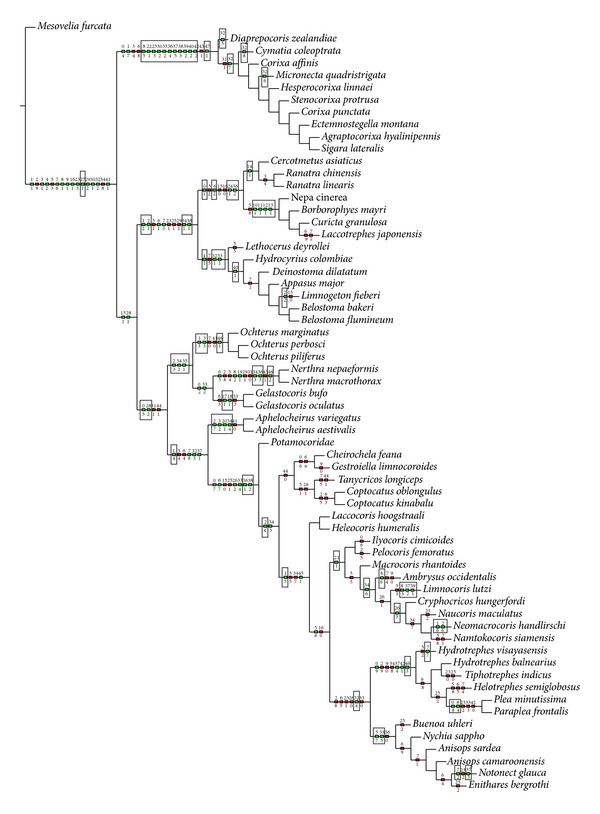
The most parsimonious tree resulting from the heuristic search with characters as equally weighted (tree length = 198, consistency index = 71, retention index = 92). A green box indicates nonhomoplasy; a red box indicates homoplasy. The number above the branch line refers to the number of a character; the number below the line of the branch refers to the number of the state of a character. The slow option is used (apomorphies and plesiomorphies together are shown).

**Figure 6:**
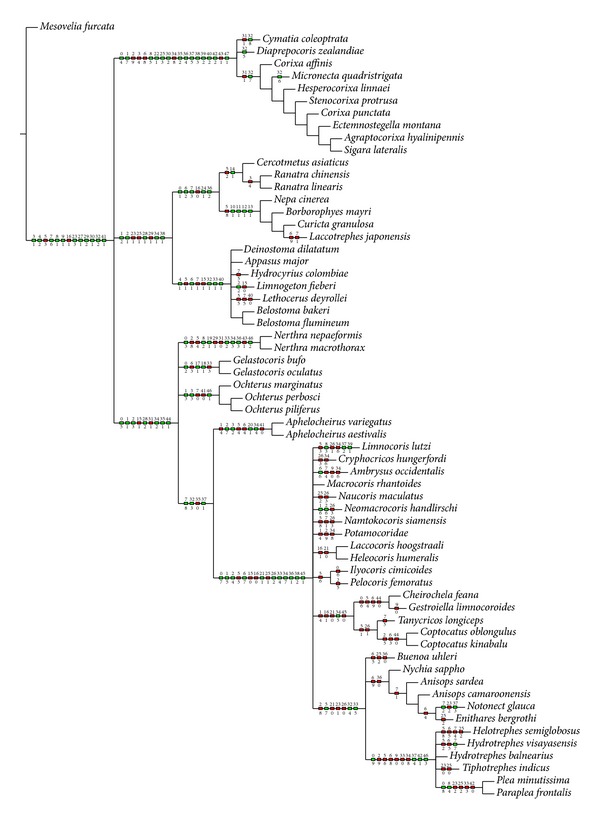
The strict consensus tree created from 100 parsimonious trees resulting from the heuristic search with characters treated as unordered and equally weighted (tree length = 221, consistency index = 63, retention index = 74). A green box indicates nonhomoplasy; a red box indicates homoplasy. The number above the branch line refers to the number of a character; the number below the line of the branch refers to number of the state of a character. The slow option is used.

**Figure 7:**
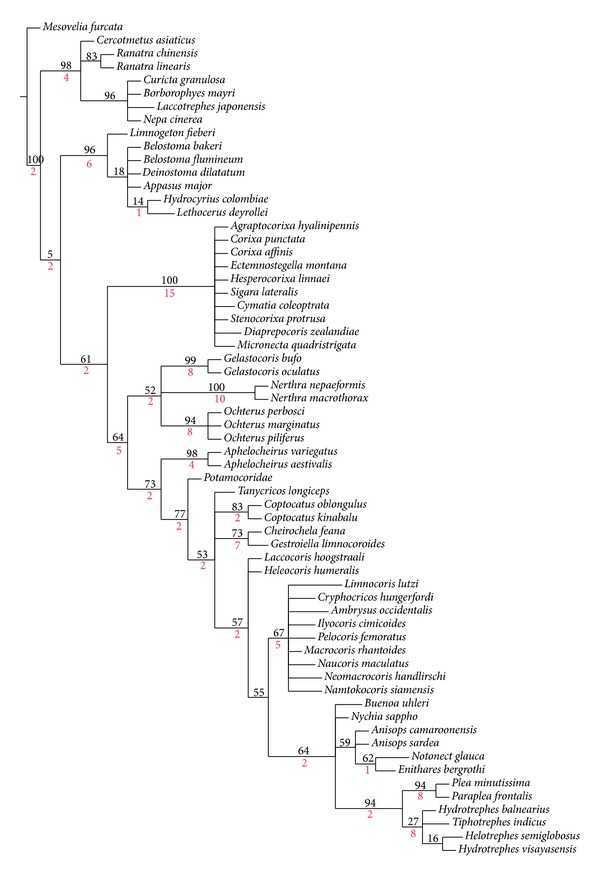
Bootstrap support; a consensus tree based on 1000 replicate samples of the character dataset, showing the bootstrap support for clades. L = 212, Ci = 62, Ri = 77. Bremer support values are marked with the red color.

The heuristic search strategy yielded 100 parsimonious trees, 199–98 steps long and with the consistency index = 72 and retention index = 92. Two of the shortest trees (198 steps long) (Figures 4 and 5) and consensus tree (Figure 6, 221 steps long) with the complete mapping of all morphological characters as nonhomoplasious = syn(apomorphies) and homoplasious represent the hypothesis with reference to the relationship within the Nepomorpha given below. The most parsimonious tree with branch support (bs = 1 for 15 in individual branches) values [66] is shown in Figure 7. The bootstrap analysis of morphological characters is also shown in Figure 7.

This infraorder represents a monophyletic taxon, which is supported by one syn(apomorphy) character (27-1; absence of the elongated plate sensillum). In this tree (Figure 4) the first step leads to the upper branch of the infraorder, to the superfamily of the Nepoidea, and the lower branch represents the remaining taxa. The Nepoidea are recognized on the basis of three synapomorphies (1-3), (28-1), and (38-1) and represent the most basal group consisting of two families: the Belostomatidae and the Nepidae. The Belostomatidae show three synapomorphies: (4-1), (32-1), (33-1), and the Nepidae: (0-1), (24-1), and (36-2). For the Belostomatinae subfamily one synapomorphy is indicated: (40-1). Moreover, in* Limnogeton fieberi* one autapomorphy (2-2) is indicated; however, for the subfamily Lethocerinae (*Lethocerus deyrollei*) the autapomorphic character is not found. The Nepinae are supported by four synapomorphies: (10-1), (11-1), (12-1), and (13-1) while in the case of the Ranatrinae one synapomorphy is visible: (14-1). These characters provide a monophyletic status for the above mentioned taxa and indicate the relationships of the sister groups Nepidae + Belostomatidae as well as two such sister clades as Nepinae + Ranatrinae (Nepidae) and Belostomatinae + Lethocerinae (Belostomatidae).

With respect to the first step, the lower branch indicates the synapomorphy (31-1) for the Corixoidea, Ochteridae, Gelastocoridae, Aphelocheiridae, Potamocoridae, Naucoridae, Notonectidae, Pleidae, and Helotrephidae.

The next branch with several (13) synapomorphies (8-5), (22-1), (25-3), (30-2), (32-7), (35-2), (36-4), (37-5) (38-3), (39-2), (40-2), (42-2), and (47-1) indicates the monophyly of Corixoidea. The monophyly of the Corixidae including the Corixinae (*Corixa affinis, Corixa punctata, Agraptocorixa hyalinipennis, *and* Hesperocorixa linnaei*) and Stenocorixinae (*Stenocorixa protrusa*) (except for Cymatiainae (*Cymatia coleoptrata*)) is supported by one synapomorphy (32-7). Within the Corixoidea, autapomorphies are estimated for the Diaprepocoridae (32-5), Micronectidae (32-6), and Cymatiainae (32-8). The subsequent branch with a synapomorphy (28-2) leads to several groups except for the Nepoidea and Corixoidea. The monophyly of the lineage Ochteridae + Gelastocoridae (Ochteroidea) is supported by 3 unambiguous synapomorphies (2-3), (34-2), and (35-1). Of these three, only (35-1) is a compelling synapomorphy of these families. Characters (2-3) and (34-2) should be indicated as synapomorphies between the Ochteridae and Gelastocorinae. Due to the fact that the Gelastocoridae (Gelastocorinae + Nerthrinae) are supported by an unambiguous synapomorphy (1-1), shared characters (2-3) and (34-2) are difficult to interpret. The monophyly of the lineage of the Nerthrinae is supported by 5 unambiguous synapomorphies (8-2), (19-1), (34-3), (36-3), and (46-2), while the lineage of the Gelastocorinae is characterized by three synapomorphies (0-2), (17-1), and (18-1). On the next branch, characters (7-8), (32-3), and (37-1) are not convincing because they are not found in all of the following taxa. The Aphelocheiridae are hypothesized to be monophyletic on the basis of 3 unambiguous synapomorphies (3-2), (20-1), and (34-4). The indicated characters (36-1) and (38-2) are convincing for the Potamocoridae, Naucoridae, Notonectidae, Pleidae, and Helotrephidae as they are uniformly present among their members. The Potamocoridae are poorly diagnosed presently and no evident characters are visible. In this reconstruction of characters the Naucoridae are hypothesized to be monophyletic based on two unambiguous synapomorphies (2-4) and (33-4); however, (33-4) is the most convincing one as it is present in all tested species (visible only in fast/slow option). The remaining different characters are spread across individual species of these families. The subfamily of Cheirochelinae is a monophyletic group on the basis of synapomorphic characters (34-5). The monophyly of Naucorinae on this cladogram is not obvious because only some species bring two synapomorphies: (21-1) and (26-3—this character is visible in the function of slow optimization). Within this subfamily, two autapomorphies (1-6) and (2-6) have been found in* Neomacrocoris handlirschi*.

The sister group relationship of the Limnocorinae and the Cryphocricinae is supported by an unambiguous synapomorphy (34-6). The monophyly of the Limnocorinae (*Limnocoris lutzi*) is characterized by three autapomorphies: (8-3), (37-2), and (39-1). Within the Cryphocricinae one autapomorphy (6-6) is indicated for* Ambrysus occidentalis*.

The monophyly of the lineage that includes the Notonectidae and the Pleidae + Helotrephidae is supported by one synapomorphy (32-4). Two synapomorphies, that is, (5-7) and (33-5), have been found for the Notonectidae (Notonectoidea). The monophyly of the superfamily Pleoidea (Pleidae and Helotrephidae) is supported by two unambiguous synapomorphies: (37-4) and (46-3). Each family also brings an individual synapomorphy: (8-4, Pleidae) and (42-1, Helotrephidae).

In this tree topology, the most (super/sub)families are found to be monophyletic; on the basis of the present data only the family of Potamocoridae (*Potamocoris nieseri*) is problematic, as no autapomorphy has been found.

The second equally parsimonious tree (Figure 5) hypothesizes the monophyly of the Nepomorpha and also finds sister relationships among most taxa in a similar way as in Figure 4. A major difference with respect to the previous tree (Figure 4), obtained also under equal weights, is the position of the Corixoidea. They are placed as a basal taxon instead of the Nepoidea (Figure 5). The most synapomorphies and autapomorphies marked in black box are the same as in the reconstruction discussed above (Figure 4).

The ambiguity in relationships among nepomorphan taxa are illustrated in the consensus tree (Figure 6). The unresolved relationships among the some species are pointed within the Corixoidea and Belostomatidae. Also the unresolved relationships are visible among the Ochteridae, Nerthrinae, and Gelastocorinae and among subfamilies of the Naucoridae (Laccocorinae, Limnocorinae, Cryphocricinae, and Naucorinae) and Potamocoridae. The polytomies are also visible among the species of Notonectidae and Helotrephidae.

Generally, the bootstrap analysis is seldom used for morphological analyses; however, its use for the purpose of the present study seems to be necessary. In 100 parsimonious trees with the same parameters (L, CI, and RI), the nodes change within the range of the analyses, mainly regarding the positions of the Corixoidea and the Nepoidea. Further nodes in terminal taxa are slightly changing and most of those nodes have very low Bremer values, suggesting little or no confidence in the groupings. The higher values of Bremer support have been calculated for the Corixoidea (Bremer = 15), while the remaining taxa have lower Bremer values (Figure 7).

The bootstrap analysis (Figure 7) shows that the character of dataset is robust with regard to the hypothesis of the monophyly of the Nepomorpha (i.e., the clade is found in 100% of the trees). The basal group of the Nepoidea is indicated in 87% of the trees, while the Nepidae is found in 98% and Belostomatidae in 96%, respectively. Those high rates of support are also maintained for the subfamilies Ranatrinae: 84%, Nepinae: 96%, and Belostomatinae: 69%. The Corixoidea, placed as a sister groups with respect to the remaining nepomorphans, is evaluated in 61% of these trees. The clade Corixoidea has received 100% support in these trees, although individual families are found in 50% of the trees.

Other taxa, except for the Nepoidea and the Corixoidea, are visible in 64% of these trees. Nonetheless, there is a weak bootstrap support (slightly above 50% of the bootstrap trees) in this dataset for a sister group relationship between the Ochteridae and the Gelastocoridae. The clade Nerthrinae + Gelastocorinae is hypothesized to be monophyletic in 44% of the trees, a fairly low bootstrap value that reflects the unstable position of the Nerthrinae in the equally parsimonious trees. The sister group relationship between the Ochteroidea and the Aphelocheiridae is found in 73% of the trees, and a relatively high bootstrap value of 77% also supports this relationship for the Potamocoridae. However, lower bootstrap support values (53-57-55% in three nodes) have been received for the Naucoridae. Nevertheless, the clade Cheirochelinae is found in 63% of the trees, the Laccocorinae in 50%, and three other of subfamilies in 67% of these trees. A sister group relationship between the Helotrephidae + Pleidae and the Notonectidae is found in 64% of the trees. A sister group relationship between the Helotrephidae + Pleidae is found in 94% of the trees and reflects the unambiguous phylogenetic position of this clade in the equally parsimonious trees. Moreover, the relationships within the Notonectidae evaluated in 83%–50% of the bootstrap trees are robustly supported in this dataset.

There is no bootstrap support (attainment of the 50% bootstrap level) for the hypothesized relationships between the representatives of* Macrocoris, Limnocoris, Ambrysus, Cryphocricos, Naucoris, Neomacrocoris,* and* Namtokocoris* as well as the helotrephid species in this dataset, even though the relationships among them are consistent in all the shortest trees. This lack of bootstrap support, in contrast to the consistent placement of these taxa in the shortest trees, reflects the fact that relatively few, but highly consistent, characters support the nodes.

## 4. Discussion

### 4.1. The Main Phylogenetic Hypothesis of Relationships within the Nepomorpha (to Be Presented in a Planned Discussion)

Previously, the phylogeny of the Nepomorpha was discussed on the basis of various morphological criteria used by China [21], Popov, [25], Rieger [26], and Mahner [10] and the hypotheses proposed by them brought about several different solutions.

Recent hypotheses regarding the relationships among taxa within the Nepomorpha based on rigorous cladistic assumptions and on molecular and morphological studies have been proposed by Hebsgaard et al. [27], and hypotheses based on molecular studies have been proposed by Hua et al. [28] and Li et al. [29]. Phylogenetic analysis of Hebsgaard et al. [27] was generally congruent with the traditional classification of Mahner [10]; however, a new superfamily of the Aphelocheiroidea (Aphelocheiridae + Potamocoridae) was evaluated, and moreover the Naucoroidea were restricted to only one family (Naucoridae) and the Ochteroidea received a new position (Ochteridae + Gelastocoridae). According to Hebsgaard et al. [27], the system of classification of the Nepomorpha included seven monophyletic superfamilies, namely, (Nepoidea, Corixoidea, Aphelocheiroidea, Naucoroidea, Ochteroidea, Notonectoidea, and Pleoidea (Pleidae + Helotrephidae)). A revised (or suggested) classification of the Nepomorpha by Hebsgaard et al. [27] based on a molecular dataset (genome) found support for just five superfamilies in the new distribution of these taxa (Corixoidea + ((Naucoroidea + Notonectoidea) + (Ochteroidea + Nepoidea). Two superfamilies from Hebsgaard et al. [27] system were lost; the Pleoidea (Pleidae + Helotrephidae) was placed in the new infraorder of the Plemorpha, while the Aphelocheiroidea sensu Hebsgaard's et al. were included into the Naucoroidea sensu Hua et al. [29].

Li et al. [29], on the basis of four Hox genes, supported the monophylies of the Nepomorpha, Naucoroidea (Aphelocheiridae + Naucoridae), Nepoidea (Belostomatidae + Nepidae), Ochteroidea (Ochteridae + Gelastocoridae), and Pleoidea (Pleidae + Helotrephidae); the Ochteroidea were the most basal lineage; the Notonectoidea contained Notonectidae only and formed a new sister relationship with the (Pleoidea + Naucoroidea) and the sister relationship with (Nepoidea + Corixoidea).

The above presented phylogenetic analyses resulted in totally different hypotheses regarding the Nepomorpha; therefore future studies in this field seem necessary.

### 4.2. How the New Values for Phylogeny and Classification System of the Nepomorpha Represent the Dataset Concerning Mouthparts and Labial Sensilla Structures?

Presently, the monophyly of the Nepomorpha is supported by an unambiguous synapomorphy (lack of the elongated plate sensillum on the labium (27-1)). This hypothesis is concordant with that of Popov [25], Mahner [10], Hebsgaard et al. [27], and Li et al. [29] but contradictory to the views of Hua et al. [28], who treated the Nepomorpha as a monophyletic group, excluding the Pleoidea.

Problems with the relationships of families (or superfamilies) represented by the 62 species in the present analysis seem far more extensive and complicated. The two most parsimonious trees shown in Figures 4 and 5 provide distinctly different solutions.

### 4.3. Relationships of (Super)families

The present system of relationships among taxa demonstrated in the first tree (Figure 4) alludes to most of the previous hypotheses [10, 25–27] with respect to the basal position of the superfamily Nepoidea (Belostomatidae and Nepidae). Presently, the superfamily is supported by three unambiguously optimized synapomorphic characters and each family is also strongly evaluated through optimized characters (i.e., the Nepidae by three characters and the Belostomatidae by four characters). In addition, the subfamilies (Nepinae and Ranatrinae) are regarded as monophyletic groups in contrast to the estimations provided by Hebsgaard et al. [27] and Mahner [10], which indicated the paraphyletic characters of the Nepinae. The next position in the presented system of relationships treats the Corixoidea as a sister group of the remaining taxa of the nepomorphans. For them, three unambiguously optimized synapomorphic characters have been found. Three families are listed within this taxon and each of them is characterized by one autapomorphy. Such an arrangement of the Corixoidea (with one family Corixidae) finds support in the studies of Popov [25], Mahner [10], and Hebsgaard et al. [27]. Then, the superfamily Ochteroidea (Ochteridae and Gelastocoridae) is positioned bellow the Corixoidea, however, in a different position from the one it had in the cladogram developed by Hebsgaard et al. [27]. In several other studies, the Ochteroidea have been placed in various positions across the system of classification. The present result confirms the hypothesis proposed by Popov [25] and Mahner [10]. The Aphelocheiridae and Potamocoridae are positioned in a similar way to the final tree (Figure 23; Hebsgaard et al. [27]); however, the superfamily Aphelocheiroidea (sensu Hebsgaard et al. [27]) presently is not formed. The Aphelocheiridae are a sister group to the Potamocoridae as a separate branch. At next step, the Potamocoridae are located separately as a sister group to the Naucoridae. Previous studies, mainly by Rieger [26], indicated a close relationship between the Potamocoridae and the Naucoridae; however, Popov [25] and Mahner [10] found a relationship between the Potamocoridae and a clade Naucoridae + Aphelocheiridae. Presently, the Naucoridae are regarded as a monophyletic family (one synapomorphy has been found) also confirmed by the studies of Hebsgaard et al. [27]. As for the remaining groups in the tree (Figure 4), namely, the Notonectidae, Helotrephidae, and Pleidae, their relationships are reminiscent of the relationships indicated by Hebsgaard et al. [27]. Actually, the clade Helotrephidae + Pleidae (Pleoidea) is also a sister group to the Notonectidae (Notonectoidea).

The classification system and the relationships among super(families) of the Nepomorpha with the Ochteroidea as the basal lineage and the configuration of the clade Pleoidea + Naucoroidea as well as the clade Nepoidea + Corixoidea presented by Li et al. (2012) are totally different from the current data (Figures 4, 5, and 6) and the previous study by Hua et al. [28], Hebsgaard et al. [27], Manher [10], and Rieger [26]. It ought to be emphasized that the Ochteroidea as a basal group has been indicated only by China [21]. Nevertheless, Popov [25] suggested that the Nepomorpha could derive from ochterid-like ancestors but that they rather derived from saldid-like stock. However, on the basis of detailed studies of the comparative morphology of the families of the Nepomorpha Parsons [22, 48, 77] suggested that the Ochteridae and Gelastocoridae were more specialized.

### 4.4. Relationship Nepomorpha in the Groups of Taxa

The consideration of the relationships presented in the cladogram (Figures 4 and 5) in wider range of comparisons among the families yields interesting results. Essentially, in the three cladograms there is a visible group of families (Ochteridae, Gelastocoridae, Aphelocheiridae, Potamocoridae, Naucoridae, Notonectidae, Helotrephidae, and Pleidae) supported by one synapomorphy (the pit chemoreceptive sensillum is present in the mentioned taxa) which correspond to the group Tripartita previously indicated by Manher [10]. This group was found in the morphological analysis an in the simultaneous morphological and molecular analysis conducted by Hebsgaard et al. [27] but it was not supported by the same molecular data. The present study also strongly supports a group composed of the families Aphelocheiridae, Potamocoridae, Naucoridae, Notonectidae, and Pleidae + Helotrephidae based on three synapomorphies. These families correspond to the group Cibariopectinata distinguished by Mahner [10] as well as to the Cibariopectinata composed of a polytomy of the clades Potamocoridae, Aphelocheiridae + Naucoridae, and Notonectidae + Pleoidea (Pleidae + Helotrephidae) presented only in the morphological analysis by Hebsgaard et al. [27]. Presently obtained data with respect to the Notonectidae as a sister group to the Pleidae + Helotrephidae (Pleoidea) are congruent with previously obtained data reported by Hebsgaard et al. [27], Manher [10], Rieger [26], Popov [25], and China [21] except for the concepts proposed by Hua et al. [28] and Li et al. [29].

### 4.5. Taxonomic and Phylogenetic Placement of Corixoidea

With regard to the tree in Figure 5, there is substantial congruence among the results of the present analyses and some hypotheses of relationships proposed by Hua et al. [29]. These include the essential points in the phylogenetical estimation of the Nepomorpha. The basal position of the Corixoidea (Figure 5) diagnosed according to the present analyses resembles the results of analyses obtained by Hua et al. [28]. Nonetheless, two points of obvious ambiguity distinguish these analyses: according to Hua et al. [28] the Notonectidae are placed as the sister group to the Aphelocheiridae + Naucoridae, whereas in the present tree (Figure 5) the distribution of the remaining taxa corresponds to the tree in Figure 4 (Aphelocheiridae, Potamocoridae, Naucoridae, Notonectidae + (Pleidae + Helotrephidae)). Moreover, as has already been mentioned above, according to Hua et al. [28] the Pleoidea do not belong to the Nepomorpha.

The present placement of the Corixoidea is different from previous several hypotheses. As for Hua et al. [28], they stated that in their results the Corixoidea was always the most basal taxon within the Nepomorpha, whereas presently the Corixoidea in the basal position is estimated only in 40% of the trees.

The aberrant morphology of the Corixidae (Corixoidea) has puzzled phylogeneticists and hence several different hypotheses have been developed about the place of the Corixidae in the systematics. Börner [78] proposed a separate division of the Sandaliorrhyncha family. However, it is now well established that the Corixidae belongs to the Nepomorpha. Both Parsons [22] and Popov [25] indicated a divergence of the Corixidae after the Nepoidea in their phylogenetic dendrograms and they stated that that group was very advanced developmentally and represented many apomorphic states. The evidence pointing to derived characters of corixoids is significant and several examples can be cited. The triangular-shaped labium is an evolutionary novelty in this group; however, it derived from the tubular four-segmented labium of other ancestral nepomorphans. In turn, the mandibles of corixid bugs shared a common pattern with other water bugs [79], but Brożek [57] pointed out similarities in the mandibles of the corixids only with respect to the mandibles of the Gelastocoridae. Moreover, the structure of the maxillary stylet in corixids was their specific characteristics, not encountered elsewhere. Characteristics of the internal structure of the mouthparts show a similar type across the Nepomorpha, indicating that the Corixoidea belongs to this infraorder. In most nepomorphan taxa the sensilla are placed along the long axis of the labium, while in the Corixoidea (except for the Cymatiainae) these sensilla are placed in the transverse bands on the labium. Different types of contrast in the distribution of sensilla on the surface of the labium can be noticed between the Corixoidea and the remaining nepomorphan families. There exists a transverse pattern of distribution of the sensilla and an autapomorphy in the case of the Corixoidea (except for the Cymatiainae). Many other modified body structures of the Corixoidea have also reached a new level of adaptation among nepomorphan taxa, and therefore they represent an advanced systematic position contrary to the suggestion of Hua et al. [28].

### 4.6. Unresolved Ancestral Nodes in the Consensus Tree

The consensus tree (Figure 6) formed on the basis of 100 parsimonious trees shows poorly resolved ancestral nodes leading to the divergence into respective taxa. This indicates that there is a substantial degree of disagreement among the trees regarding individual parameters, although the characters/characters states have been weighed. This especially refers to two groups: the Nepoidea and the Corixoidea; the positions of these taxa are a major problem. Moreover, an unsatisfactory degree of relationships is also evident in the group of naucorids. Unresolved relationships are shown between many naucorid species and the Potamocoridae.

In addition, the superfamily Ochteroidea has been spread out over three polytomous taxa: the Ochteridae, Gelastocorinae, and Nerthrinae.

It can be expected that in future cladistic studies taking into account a wider range of morphological characters will stabilize the positions of most clades that have been recognized so far.

The first step towards achieving such goal can be combining the characters from the present matrix with the morphological matrix used by Hebsgaard et al. [27]. However, it would require further work on a number of significant features so that they would correspond with the list of species that have already been studied.

## 5. Conclusion

- The present study supports the monophyly of the Nepomorpha and the monophyly of all currently recognized families. A slight modification in the systematic classification of families of the Nepomorpha is proposed (Figure 4): ((Nepidae + Belostomatidae), (Diaprepocoridae + Corixidae + Micronectidae), (Ochteridae + Gelastocoridae), Aphelocheiridae, Potamocoridae, Naucoridae, Notonectidae, and (Pleidae + Helotrephidae)).
- The present hypothesis concurs with Popov [25], Mahner [10], and Hebsgaard et al.'s [27], in the placement of the Nepoidea (Nepidae + Belostomatidae) and Corixoidea (Diaprepocoridae + Corixidae + Micronectidae) as a sister group with respect to the remaining nepomorphan families but differs in the placement of the Potamocoridae.
- The Potamocoridae is recognized as a sister group to the Naucoridae (Figure 4) and they together form the superfamily Naucoroidea (Naucoridae + Potamocoridae) (Figure 6). This issue remains open for further investigation.
- Presently is identified that the superfamily Aphelocheiroidea includes only one family, the Aphelocheiridae, in contrast to hypothesis of Hebsgaard et al.'s [27], that the Aphelocheiroidea consists of the Aphelocheiridae + Potamocoridae.
- The seven superfamilies of the Nepomorpha are confirmed on the basis of the available dataset: the Nepoidea ((Nepidae + Belostomatidae), Corixoidea (Diaprepocoridae + Corixidae + Micronectidae), Ochteroidea (Ochteridae + Gelastocoridae), Aphelocheiroidea (Aphelocheiridae), Naucoroidea (Potamocoridae + Naucoridae), Notonectoidea (Notonectidae), and Pleoidea (Pleidae + Helotrephidae)).
- Information on structures obtained across the analyzed dataset indicated that the group of corixids displayed 13 autapomorphies (more than in other nepomorphan taxa) indicating their strong apomorphic forms and their advanced position in the system of classification.
- The analysis has revealed five autapomorphies in the dataset for the Nerthrinae with respect to two synapomorphies in the Gelastocorinae. Both subfamilies are monophyletic. According to Cassis and Silveira [53] the Nerthrinae is monophyletic (it refers to the* alaticollis* species group). It would be interesting to investigate further these morphological diversities in future phylogenetic studies focusing on the Gelastocoridae and elevating the rank of the Nerthrinae to family level. Cassis and Gross [80] admitted that the Nerthrinae is the more diverse of the two subfamilies of the Gelastocoridae.
- In the present study, close relationships among families and/or at the superfamilies level of the Nepomorpha which are presented in Figure 4 find confirmation in other morphological hypotheses of the phylogeny. The concurrence encompasses mainly the hypotheses of Popov [25] and Mahner [10]. Only four family relationships indicated by Hebsgaard et al.'s [27] hypothesis are concurrent with the current data. The relationships of nepomorphan families inferred from the present morphological study and the relationships based only on molecular data evaluated by other authors do not show strong mutual support. Presently, only the Corixoidea at the basal position shown in Figure 5 can be inferred as the sister group to the remaining nepomorphans like the genetic thesis proposed by Hua et al. [28].

An essential difference between the present paper and the remaining publications of Brożek ([57–59], 2014 in press) is that in the present paper the focus is on establishing the relationships among the families of the Nepomorpha and their classification, whereas in my previous papers the main objectives were to describe new morphological characters of the mouthpart of Nepomorpha and to provide their detailed documentation using SEM images and some schematic line drawings. In the previous papers only a preliminary estimation of these characters based on the ground plan was conducted, attempting to suggest the relationships among the nepomorphan families.

New achievements of the present paper in comparison to previous publications are listed below.

- The paper presented a description and documentation of the presence and distribution of the mechanosensilla on the labium in the Mesoveliidae that were selected as the basal families of the Gerromorpha and an outgroup for the Nepomorpha.
- On the basis of the cladistic method all characters of the mouthparts were polarized in relation to the outgroup.
- The new dataset for mouthparts was displayed in the matrix form.
- Several characters were developed for the family Potamocoridae on the basis of data accessed from scientific references in order to compile characters for the analysis of all 13 families of the Nepomorpha.
- The phylogenetic estimation of the morphological characters was conducted with the aid of computer programs used for cladistic analysis.
- The relationships among families and subfamilies of the Nepomorpha were presented on phylogenetic trees.
- A new system of relationships and classification of the Nepomorpha was proposed in relation to previous hypotheses of other authors based on the cladistic analysis of morphological characters of the mouthparts (stylets bundle, sense organ of labium, and labial segments).

## Figures and Tables

**Table 1:** The list of fifty-six nepomorphan species which have been studied. Additionally, one species of the outgroup (Gerromorpha: Mesoveliidae: *Mesovelia furcata*) has been included.

Families	Subfamilies	Species	Authors
Mesoveliidae	Mesoveliinae	*Mesovelia furcata *	Mulsant and Rey, 1852

Nepidae	Nepinae	*Curicta granulosa *	De Carlo, 1951
		*Borborophyes mayri *	Stål, 1871
		*Laccotrephes japonensis *	(Scott, 1874)
		*Nepa cinerea *	Linnaeus, 1758
	Ranatrinae	*Cercotmetus asiaticus *	Amyot & Serville, 1843
		*Ranatra chinensis *	(Mayr, 1865)

Belostomatidae	Belostomatinae	*Belostoma flumineum *	Say, 1832
		*Deinostoma dilatatum *	(Say)
		*Appasus major *	(Esaki, 1934)
		*Hydrocyrius colombiae *	Spinola, 1850
		*Limnogeton fieberi *	Mayr, 1853
	Lethocerinae	*Lethocerus deyrollei *	(Vuillefroy, 1864)

Ochteridae		*Ochterus marginatus *	(Latreille, 1804)
		*Ochterus piliferus *	Kormilev 1973

Gelastocoridae	Gelastocorinae	*Gelastocoris oculatus *	(Fabricius, 1798)
	Nerthrinae	*Nerthra nepaeformis *	(Fabricius, 1798)
		*Nerthra macrothorax *	(Montrouzier, 1855)

Aphelocheiridae		*Aphelocheirus variegatus *	Kiritschenko, 1925
		*Aphelocheirus aestivalis *	(Fabricius, 1794)

Naucoridae	Cheirochelinae	*Cheirochela feana *	Montandon, 1897
		*Gestroiella limnocoroides *	Montandon, 1897
		*Coptocatus oblongulus *	Montandon, 1909
		*Coptocatus kinabalu *	Polhemus D. 1986
		*Tanycricos longiceps *	La Rivers, 1971
	Laccocorinae	*Laccocoris hoogstraali *	La Rivers, 1970
		*Heleocoris humeralis *	Signoret, 1861
	Limnocorinae	*Limnocoris lutzi *	La Rivers, 1957
	Cryphocricinae	*Cryphocricos hungerfordi *	Usinger,1947
		*Ambrysus occidentalis *	La Rivers, 1951
	Naucorinae	*Ilyocoris cimicoides *	(Linnaeus 1758)
		*Pelocoris femoratus *	(Palisot de Beauvois 1820)
		*Macrocoris rhantoides *	Bergroth
		*Naucoris maculatus *	Fabricius, 1798
		*Neomacrocoris handlirschi *	(Montandon, 1909)
		*Namtokocoris siamensis *	Sites 2007

Pleidae		*Paraplea frontalis *	(Fieber, 1844)

Helotrephidae	Helotrephinae	*Helotrephes semiglobosus *	Stål, 1860
		*Hydrotrephes visayasensis *	Zettel, 2002
		*Hydrotrephes balnearius *	(Bergroth, 1918)
		*Tiphotrephes indicus *	(Distant, 1910)

Notonectidae	Anisopinae	*Anisops camaroonensis *	Signoret
		*Anisops sardea *	Herrich-Schäffer 1849
		*Buenoa uhleri *	Truxal, 1953
	Notonectinae	*Notonecta glauca *	Linnaeus, 1758
		*Enithares bergrothi *	Montandon, 1892
		*Nychia sappho *	Kirkaldy, 1901

Corixidae	Corixinae	*Agraptocorixa hyalinipennis *	(Fabricius, 1803)
		*Corixa punctata *	(Illiger, 1807)
		*Corixa affinis *	Leach, 1817
		*Ectemnostegella montana *	Lundblad, 1928
		*Hesperocorixa linnaei *	(Fieber, 1848)
		*Sigara lateralis *	(Leach, 1817)
	Cymatiainae	*Cymatia coleoptrata *	(Fabricius, 1777)
	Stenocorixinae	*Stenocorixa protrusa *	Horváth, 1926

Diaprepocoridae		*Diaprepocoris zealandiae *	Hale, 1924

Micronectidae		*Micronecta quadristrigata *	Breddin, 1905

Potamocoridae		*Potamocoris nieseri *	van Doesburg, 1984 [18]

**Table 2:** New set of characters of the mouthparts—stylets bundle, sense organs, and labial segments of the Nepomorpha.

Number of characters	State of characters
K0. Mandibular file	(0) Evenly serrated (short spines) and medium length; *Mesovelia furcata, *Belostomatinae
	(1) Evenly serrated (short spines) and short length; Nepidae
	(2) Evenly grooved (blunt pegs) and medium length; Gelastocorinae
	(3) Evenly grooved (blunt pegs) and long; Nerthrinae
	(4) Evenly grooved (massive plates) and medium length; Corixoidea
	(5) Unevenly serrated (short and long spines) and medium length;
	Ochteridae and Aphelocheiridae
	(6) Unevenly serrated (blunt pegs, short and long spines) and long; Cheirochelinae: *Cheirochela feana* and *Gestroiella limnocoroides *
	(7) Unevenly serrated (blunt pegs and long spines) and medium length; *Tanycricos longiceps, Laccocoris hoogstraali, Helocoris humeralis, Pelocoris femoratus, Ambrysus occidentalis, Cryphocricos hungerfordi, Macrocoris rhantoides, Neomacrocoris handlirschi, Limnocoris lutzi, *and Notonectidae
	(8) Unevenly serrated (plaques, nodule and long spines) and long; Pleidae
	(9) Unevenly serrated (blunt pegs, short spines, nodule, long spines) and long: Helotrephidae
	(?) Lack of data; Potamocoridae*, Coptocatus oblongulus, Coptocatus kinabalu, Naucoris maculatus, Namtokocoris siamensis *

K1. Shapes of the apices of the maxillae	(0) Symmetrical apices (both apices straight, slightly narrow and flat); *Mesovelia furcata *
	(1) Symmetrical apices (both apices straight and narrow); *Gelastocoris oculatus, Nerthra nepaeformis, N. macrothorax *
	(2) Asymmetrical apices (the right one straight, the left one with a narrow lobe); Nepidae and Belastomatidae
	(3) Asymmetrical apices (the right one curved, the left one straight and narrow); *Ochterus marginatus, O. perbosci *
	(4) Asymmetrical apices (the right one straight and narrow, the left one wide and curved), Aphelocheiridae, Potamocoridae, *Coptocatus oblongulus, Coptocatus kinabalu, Cheirochela feana,* and *Gestroiella limnocoroides *
	(5) Asymmetrical apices (the right one straight and tapered, the left one lancet-shaped); *Laccocoris hoogstraali, Helocoris humeralis, Ilyocoris cimicoides, Pelocoris femoratus, Ambrysus occidentalis, Cryphocricos hungerfordi, Macrocoris rhantoides, Naucoris maculatus, Namtokocoris siamensis, Limnocoris lutzi, *Notonectidae, Pleidae, and Helotrephidae
	(6) Asymmetrical apices (the right one straight and tapered, the left one cap-like) *Neomacrocoris handlirschi *(Naucorinae)
	(7) Asymmetrical apices (the right one flat and blunt, the left one sharp, long and hooked); Corixoidea
	(?) Lack of data*; Gelastocoris bufo, Ochterus piliferus, Tanycricos longiceps *

K2. Rupturing device	(0) Exposed; the dorsal external (brdex) and internal bristles (brdin) and ventral external (brvex) and internal bristles (brvin) densely arranged in rows along the edges of the maxillae; *Mesovelia furcata *
	(1) Exposed; external and internal bristles in rows along the ventral and dorsal edges of the maxillae, stiff bristles (brvex) sparsely arranged and separated from one other;* Belostoma flumineum, Deinostoma dilatatum, Appasus major, Hydrocyrius colombiae, Lethocerus deyrollei, Curicta granulosa, Borborophyes mayri, Laccotrephes japonensis, Nepa cinerea, Cercotmetus asiaticus, Ranatra chinensis, R. linearis*.
	(2) Exposed; external and internal short spines (spvex, spdex, spvin); *Limnogeton fieberi *
	(3) Exposed; dorsal bristles (brdex) very short; Ochteridae and Gelastocorinae
	(4) Exposed; stiff bristles (brvex) distributed along the ventral edges; *Tanycricos longiceps*, *Cheirochela feana* and *Gestroiella limnocoroides*, *Laccocoris hoogstraali, Helocoris humeralis, Ilyocoris cimicoides, Ambrysus occidentalis, Cryphocricos hungerfordi, Macrocoris rhantoides, Naucoris maculatus, Namtokocoris siamensis, *and *Limnocoris lutzi *
	(5) Exposed; dorsal bristles (brdex) smaller than the ventral ones and slightly visible;* Coptocatus oblongulus, C. kinabalu, *and *Pelocoris femoratus *
	(6) Exposed; the bristles arranged in tufts on the dorsal (brdex) and ventral (brvex) edges; *Neomacrocoris handlirschi *
	(7) Hidden; short bristles (brvex, brdex) placed flat against the insides of the maxillae; *Aphelocheiridae, Buenoa uhleri, Anisops sardea, Anisops camaroonensis, Enithares bergrothi, Notonecta glauca, *and *Nychia sappho *
	(8) Hidden; ventral bristles (brvin) inside of the maxillae; Nerthrinae,
	(9) Almost reduced; externally the maxillae totally smooth; inside, preapically, the right maxilla with seven very short spines; Corixoidea, Potamocoridae, Pleidae, and Helotrephidae
	(?) Lack of data; *Gelastocoris bufo *

K3. Cross-section of the maxillae	(0) Trapezoid-shaped maxillae with four short external processes; *Mesovelia furcata *
	(1) Dorsolaterally extended maxillae with two wide lobes (processes); most of the Nepidae (except for *Ranatra chinensis* and *R. linearis*) and all Belostomatidae, Gelastocoridae, Naucoridae, Pleidae, Helotrephidae, and Notonectidae
	(2) Ventrolaterally extended maxillae with a wide lobe; Aphelocheiridae
	(3) Pentagonal-shaped maxillae with two external lobes; Ochteridae
	(4) Oval maxillae, flattened laterally without the external lobe; *Ranatra chinensis, R. linearis, *and corixoids species
	(?) Lack of data; Potamocoridae

K4. Cross-section of the mandible	(0) Short, suboval mandibles situated between dorsal and ventral external processes and not overlapped by the maxillae; *Mesovelia furcata *
	(1) Mandibles completely surrounded by the maxillae; Belostomatidae
	(2) Mandibles only partly overlapped by the maxillae; remaining species of Nepomorpha
	(?) Lack of data; Potamocoridae

K5. Chaetica sensilla CH3	(0) Present on the III and IV segments: *Mesovelia furcata *
	(1) Present on the I, II, and III segments: *Belostoma flumineum, Deinostoma dilatatum, Appasus major, Hydrocyrius colombiae, Limnogeton fieberi, Coptocatus oblongulus, Coptocatus kinabalu, *and *Tanycricos longiceps *
	(2) Present on the IV segment: *Cercotmetus asiaticus, Ranatra chinensis, R. linearis,*and *Hydrotrephes visayasensis *
	(3) Present on the III segment: *Ochterus piliferus, O. marginatus, Gelastocoris oculatus, Limnocoris lutzi, *and Corixoidea,
	(4) Present on the I and II segments: *Nerthra nepaeformis, N. macrothorax, Aphelocheirus variegatus, A. aestivalis, Cheirochela feana, *and *Gestroiella limnocoroides *
	(5) Present on the II and III segments: *Lethocerus deyrollei, Laccocoris hoogstraali, Heleocoris humeralis, Cryphocricos hungerfordi, Ambrysus occidentalis, Macrocoris rhantoides, Naucoris maculatus, *and *Neomacrocoris handlirschi *
	(6) Present on the II, III, and IV segments: *Ilyocoris cimicoides, Pelocoris femoratus, Paraplea frontalis, Hydrotrephes balnearius, *and *Tiphotrephes indicus *
	(7) Present on the I, II, III, and IV segments: *Anisops camaroonensis, A. sardea, Buenoa uhleri, Notonecta glauca, Enithares bergrothi, *and *Nychia sappho *
	(8) Absent: *Curicta granulosa, Borborophyes mayri, Laccotrephes japonensis, Nepa cinerea, Namtokocoris siamensis, *and *Helotrephes semiglobosus *
	(?) Lack of data: Potamocoridae

K6. Chaetica sensilla CH2	(0) Present on the I and II segments: *Mesovelia furcata, Ochterus piliferus, O. marginatus, Nerthra nepaeformis, *and *N. macrothorax *
	(1) Present on the II and III segments: *Belostoma flumineum, Deinostoma dilatatum, Appasus major, Hydrocyrius colombiae, Limnogeton fieberi, *and *Lethocerus deyrollei *
	(2) Present on the IV segment: *Curicta granulosa, Borborophyes mayri, Nepa cinerea, Cercotmetus asiaticus, Ranatra chinensis, *and *R. linearis *
	(3) Present on the I and III segments: *Gelastocoris oculatus, Coptocatus oblongulus, *and *Coptocatus kinabalu *
	(4) Present on the I, II, and III segments: *Aphelocheirus variegatus, A. aestivalis, Notonecta glauca, *and *Enithares bergrothi *
	(5) Present on the II, III, and IV segments: *Helotrephes semiglobosus, Hydrotrephes visayasensis, *and *Buenoa uhleri *
	(6) Present on the I segment: *Ambrysus occidentalis *
	(7) Present on the II segment: *Tanycricos longiceps, Laccocoris hoogstraali, Heleocoris humeralis, Limnocoris lutzi, Cryphocricos hungerfordi, Ilyocoris cimicoides, Pelocoris femoratus, Macrocoris rhantoides, Naucoris maculatus, Neomacrocoris handlirschi, *and *Namtokocoris siamensis *
	(8) Present on the III segment: *Paraplea frontalis, Plea minutissima, Hydrotrephes balnearius, Tiphotrephes indicus, *and Corixoidea
	(9) Absent: *Laccotrephes japonensis, Cheirochela feana, Gestroiella limnocoroides, Anisops camaroonensis, Anisops sardea, *and *Nychia sappoho *
	(?) Lack of data: Potamocoridae

K7. Chaetica sensilla CH1	(0) Present on the I and II segments: *Mesovelia furcata, Ochterus piliferus, *and *O. marginatus *
	(1) Present on the II and III segments: *Belostoma flumineum, Deinostoma dilatatum, Appasus major, Limnogeton fieberi, Laccotrephes japonensis, Namtokocoris siamensis, Anisops camaroonensis, Anisops sardea, *and *Enithares bergrothi *
	(2) Present on the I, II, and III segments: *Notonecta glauca *
	(3) Present on the IV segment: *Curicta granulosa, Borborophyes mayri, Nepa cinerea, Cercotmetus asiaticus, *and *Ranatra chinensis *
	(4) Present on the I segment: *Ambrysus occidentalis* and *Helotrephes semiglobosus *
	(5) Present on the II segment: *Hydrocyrius colombiae, Lethocerus deyrollei, *and *Tanycricos longiceps, *
	(6) Present on the III segment: *Gelastocoris oculatus, Nerthra nepaeformis, N. macrothorax*, and Corixoidea
	(7) Present on the I and IV segments: *Hydrotrephes visayasensis *
	(8) Absent: *Aphelocheirus variegatus*, *A. aestivalis, Cheirochela feana, Gestroiella limnocoroides, Coptocatus oblongulus, Coptocatus kinabalu, Laccocoris hoogstraali, Heleocoris humeralis, Limnocoris lutzi, Cryphocricos hungerfordi, Ilyocoris cimicoides, Pelocoris femoratus, Macrocoris rhantoides, Naucoris maculatus, Neomacrocoris handlirschi, Paraplea frontalis, Plea minutissima, Hydrotrephes balnearius, Tiphotrephes indicus, Buenoa uhleri, *and *Nychia Sappoho *
	(?) Lack of data: Potamocoridae

K8. Dorsal hairs, proprioceptive sensilla; location and number	(0) Long, one pair on the dorsal side of the II segment; *Mesovelia furcata *
	(1) Short, one pair on the dorsal side of the II segment; remaining nepomorphan species
	(2) Long, three pairs on the dorsal side of the II segment; *Nerthra nepaeformis* and *N. macrothorax *
	(3) Dispersed (III pairs of various lengths); *Limnocoris lutzi *
	(4) Two pairs, short; *Paraplea frontalis* and *Plea minutissima *
	(5) Lack of proprioceptive sensillum; corixoids species
	(?) Lack of data; Potamocoridae

K9. Ventral hairs, proprioceptive sensilla; location and number	(0) One pair present on the ventral side (II segment); *Mesovelia furcata, Belostoma flumineum, Deinostoma dilatatum, Gestroiella limnocoroides, Ambrysus occidentalis, Paraplea frontalis, Plea minutissima, Helotrephes semiglobosus, Hydrotrephes visayasensis, Hydrotrephes balnearius,Tiphotrephes indicus *
	(1) Lack of proprioceptive sensillum; corixoids species and the remaining nepomorphan species

K10. Squamiforme sensillum	(0) Absent; *Mesovelia furcata* and most of the nepomorphans
	(1) Present; only in *Curicta granulosa, Borborophyes mayri*, *Laccotrephes japonensis, * and *Nepa cinerea *
	(?) Lack of data; Potamocoridae

K11. Trichobothrium sensillum	(0) Absent; *Mesovelia furcata* and most of the nepomorphans
	(1) Present; *Curicta granulosa*, *Borborophyes mayri*, *Laccotrephes japonensis, *and *Nepa cinerea *

K12. Basiconic sensillum	(0) Absent; *Mesovelia furcata* and most of the nepomorphans
	(1) Present; *Curicta granulosa*, *Borborophyes mayri*, *Laccotrephes japonensis*, and *Nepa cinerea *
	(?) Lack of data; Potamocoridae

K13. Club-like sensillum	(0) Absent; *Mesovelia* and most of the nepomorphans
	(1) Present; *Curicta granulosa*, *Borborophyes mayri*, *Laccotrephes japonensis*,* * and *Nepa cinerea *
	(?) Lack of data; Potamocoridae

K14. Paddle-like sensillum	(0) Absent; *Mesovelia furcata* and most of the nepomorphans
	(1) Present; *Cercotmetus asiaticus*, *Ranatra chinensis, *and *R. linearis *
	(?) Lack of data; Potamocoridae

K15. Cupola-shaped sensillum	(0) Absent;* Mesovelia furcata* and most of the nepomorphans
	(1) Present; *Belostoma flumineum*, *Deinostoma dilatatum*, *Appasus major*, *Hydrocyrius colombiae*,* Lethocerus deyrollei, Ochterus marginatus, Ochterus piliferus, Gelastocoris oculatus, Nerthra nepaeformis, N. macrothorax*, *Aphelocheirus variegatus, * and *A. aestivalis *
	(?) Lack of data; Potamocoridae

K16. Peg sensillum	(0) Absent; *Mesovelia furcata*,* Curicta granulosa*, *Borborophyes mayri*, *Laccotrephes japonensis*, *Nepa cinerea, Cercotmetus asiaticus*, *Ranatra chinensis, R. linearis, Limnocoris lutzi, Cryphocricos hungerfordi, Ambrysus occidentalis*, *Ilyocoris cimicoides*,* Pelocoris femoratus*, *Macrocoris rhantoides*, *Naucoris maculatus*, *Neomacrocoris handlirschi*, *Namtokocoris siamensis, Paraplea frontalis, Helotrephes semiglobosus*, *Hydrotrephes visayasensis*, *Hydrotrephes balnearius*, *Tiphotrephes indicus*, *Anisops camaroonensis, A. sardea, Buenoa uhleri, Notonecta glauca, Enithares bergrothi*, and *Nychia sappho *
	(1) Present; *Belostoma flumineum*, *Deinostoma dilatatum*, *Appasus major*, *Hydrocyrius colombiae*,* Lethocerus deyrollei, Ochterus marginatus, O. piliferus, Gelastocoris oculatus, Nerthra nepaeformis, N. macrothorax*, *Aphelocheirus variegatus, A. aestivalis, Cheirochela feana, Gestroiella limnocoroides, Coptocatus oblongulus, C. kinabalu, Laccocoris hoogstraali, Helocoris humeralis*, and corixoids species
	(?) Lack of data; Potamocoridae

K17. Finger-like sensillum	(0) Absent; *Mesovelia furcata* and most of the nepomorphans
	(1) Present;* Gelastocoris oculatus* and *G. bufo *
	(?) Lack of data; Potamocoridae

K18. Freniale-like sensillum	(0) Absent; *Mesovelia* and most of the nepomorphans
	(1) Present;* Gelastocoris oculatus* and *G. bufo *
	(?) Lack of data; Potamocoridae

K19. Chaetica sensillum with a bisected tip	(0) Absent; *Mesovelia furcata* and most of the nepomorphans
	(1) Present; *Nerthra nepaeformis, N. macrothorax *
	(?) Lack data; Potamocoridae

K20. Star-like sensillum	(0) Absent; *Mesovelia furcata* and most of the nepomorphans
	(1) Present;* Aphelocheirus variegatus* and * A. aestivalis *
	(?) Lack of data; Potamocoridae

K21. Multilobed sensillum	(0) Absent; *Mesovelia furcata* and some of nepomorphans
	(1) Present; *Limnocoris lutzi, Cryphocricos hungerfordi, Ambrysus occidentalis, Ilyocoris cimicoides*,* Pelocoris femoratus*, *Macrocoris rhantoides*, *Naucoris maculatus*, *Neomacrocoris handlirschi*, and *Namtokocoris siamensis *
	(?) Lack of data; Potamocoridae

K22. Ribbon-like sensillum	(0) Absent; *Mesovelia furcata* and most of the nepomorphans
	(1) Present; corixoids species
	(?) Lack of data; Potamocoridae

K23. Trichoid sensillum (TRS) on the dorsal side of the IV segment	(0) Present, short: *Mesovelia furcata* and *Tiphotrephes indicus *
	(1) Present, short and long: *Curicta granulosa, Borborophyes mayri, Laccotrephes japonensis, Nepa cinerea, Cercotmetus asiaticus, Ranatra chinensis, Belostoma flumineum, Deinostoma dilatatum, Appasus major, Hydrocyrius colombiae, Limnogeton fieberi, Lethocerus deyrollei, Helotrephes semiglobosus, Hydrotrephes visayasensis, H. balnearius, Anisops camaroonensis, A. sardea, Buenoa uhleri, Enithares bergrothi, *and *Nychia sappho *
	(2) Present, long: *Paraplea frontalis* and *Notonecta glauca *
	(3) Absent: *Ochterus piliferus, O. marginatus, Gelastocoris oculatus, Nerthra nepaeformis, N. macrothorax, Aphelocheirus variegatus, A. aestivalis, Cheirochela feana, Gestroiella limnocoroides, Coptocatus oblongulus, C. kinabalu, Tanycricos longiceps, Laccocoris hoogstraali, Heleocoris humeralis, Limnocoris lutzi, Cryphocricos hungerfordi, Ambrysus occidentalis, Ilyocoris cimicoides, Pelocoris femoratus, Macrocoris rhantoides, Naucoris maculatus, Neomacrocoris handlirschi (K23 invisible?), Namtokocoris siamensis*, and corixoids species
	(?) Lack of data: Potamocoridae

K24. Trichoid sensilla on the lateral side of the IV segment	(0) Absent: *Mesovelia furcata, Belostoma flumineum, Deinostoma dilatatum, Appasus major, Hydrocyrius colombiae, Limnogeton fieberi, Lethocerus deyrollei, Ochterus piliferus, O. marginatus, Gelastocoris oculatus, Nerthra nepaeformis, N. macrothorax, Aphelocheirus variegatus, A. aestivalis, Cheirochela feana, Gestroiella limnocoroides, Coptocatus oblongulus, C. kinabalu, Tanycricos longiceps, Laccocoris hoogstraali, Heleocoris humeralis, Limnocoris lutzi, Cryphocricos hungerfordi, Ambrysus occidentalis, Ilyocoris cimicoides, Pelocoris femoratus, Macrocoris rhantoides, Naucoris maculatus, Neomacrocoris handlirschi, Namtokocoris siamensis, Helotrephes semiglobosus, Hydrotrephes visayasensis, H. balnearius, Tiphotrephes indicus, Anisops camaroonensis, A. sardea, Buenoa uhleri, Notonecta glauca, Enithares bergrothi, Nychia sappho, *corixoids species, and Pleidae
	(1) Present: *Curicta granulosa, Borborophyes mayri, Laccotrephes japonensis, Nepa cinerea, Cercotmetus asiaticus, *and *Ranatra chinensis *
	(?) Lack of data: Potamocoridae

K25. Trichoid sensillum on the ventral side of the IV segment	(0) Present, short: *Mesovelia furcata, Ochterus piliferus, O. marginatus, Gelastocoris oculatus, Nerthra nepaeformis, N. macrothorax, Aphelocheirus variegatus, A. aestivalis, *and *Tiphotrephes indicus *
	(1) Present short and long: *Curicta granulosa, Borborophyes mayri, Laccotrephes japonensis, Nepa cinerea, Cercotmetus asiaticus, Ranatra chinensis, Belostoma flumineum, Deinostoma dilatatum, Appasus major, Hydrocyrius colombiae, Limnogeton fieberi, Lethocerus deyrollei, Cheirochela feana, Gestroiella limnocoroides, Coptocatus oblongulus, C. kinabalu, Tanycricos longiceps, Laccocoris hoogstraali, Heleocoris humeralis, Limnocoris lutzi, Cryphocricos hungerfordi, Ambrysus occidentalis, Ilyocoris cimicoides, Pelocoris femoratus, Macrocoris rhantoides, Naucoris maculatus, Neomacrocoris handlirschi, Namtokocoris siamensis, Hydrotrephes visayasensis, H. balnearius, Anisops camaroonensis, A. sardea, Notonecta glauca, *and *Nychia sappho *
	(2) Present, long: *Paraplea frontalis, Plea minutissima, Helotrephes semiglobosus, Buenoa uhleri, *and *Enithares bergrothi *
	(3) Absent: corixoids species
	(?) Lack of data: Potamocoridae

K26. Trichoid sensillum on the dorsal side of the third segment	(0) Absent: *Mesovelia furcata, Curicta granulosa, Borborophyes mayri, Laccotrephes japonensis, Nepa cinerea, Cercotmetus asiaticus, Ranatra chinensis, Belostoma flumineum, Deinostoma dilatatum, Appasus major, Hydrocyrius colombiae, Limnogeton fieberi, Lethocerus deyrollei, Ochterus piliferus, O. marginatus, Gelastocoris oculatus, Nerthra nepaeformis, N. macrothorax, Aphelocheirus variegatus, A. aestivalis, Paraplea frontalis, Plea minutissima, Helotrephes semiglobosus, Hydrotrephes visayasensis, H. balnearius, Tiphotrephes indicus, Anisops camaroonensis, A. sardea, Notonecta glauca, Buenoa uhleri, Enithares bergrothi, Nychia sappho, *and corixoids species
	(1) Present, short: *Coptocatus oblongulus, C. kinabalu, Tanycricos longiceps, *and *Limnocoris lutzi *
	(2) Present, short and long: *Cheirochela feana, Gestroiella limnocoroides, Laccocoris hoogstraali, Heleocoris humeralis, Ambrysus occidentalis, Pelocoris femoratus, Macrocoris rhantoides, *and *Ilyocoris cimicoides *
	(3) Present, long: *Cryphocricos hungerfordi, Naucoris maculatus, Namtokocoris siamensis, *and *Neomacrocoris handlirschi *
	(?) Lack of data: Potamocoridae

K27. Elongated plate sensillum	(0) Present; *Mesovelia furcata *
	(1) Absent: all species of Nepomorpha

K28. Pit sensilla and their distribution	(0) Pit sensillum absent; *Mesovelia furcata* and corixoids species
	(1) Pit sensillum present and localised rather laterally; all species of Nepidae and Belostomatidae
	(2) Pit sensilla placed centrally;* Ochterus piliferus, O. marginatus, Gelastocoris oculatus, Nerthra nepaeformis, N. macrothorax, Aphelocheirus variegatus, A. aestivalis, Anisops cameroonensis, A. sardea, Notonecta glauca, Buenoa uhleri, Enithares bergrothi, Nychia sappho, Cheirochela feana, Gestroiella limnocoroides, Coptocatus oblongulus, C. kinabalu, Tanycricos longiceps, Limnocoris lutzi, Laccocoris hoogstraali, Heleocoris humeralis, Cryphocricos hungerfordi, Ambrysus occidentalis, Pelocoris femoratus, Macrocoris rhantoides, Ilyocoris cimicoides, Naucoris maculatus, Namtokocoris siamensis, Neomacrocoris handlirschi, Paraplea frontalis, Plea minutissima, Helotrephes semiglobosus, Hydrotrephes visayasensis, H. balnearius, *and *Tiphotrephes indicus *
	(?) Lack of data; Potamocoridae

K29. Types and distribution of apical chemosensilla	(0) Peg sensilla placed centrally; *Mesovelia furcata *
	(1) Papillae sensilla (PAS1) distributed over the tip of the labium; all species of the Nepidae and Belostomatidae, and Nerthrinae (*Nerthra nepaeformis* and *N. macrothorax*),
	(2) Papillae sensilla (PAS2) distributed over the tip of the labium; *Ochterus piliferus, O. marginatus, Gelastocoris oculatus, Aphelocheirus variegatus, A. aestivalis, Anisops camaroonensis, Anisops sardea, Notonecta glauca, Buenoa uhleri, Enithares bergrothi, Nychia sappho, Cheirochela feana, Gestroiella limnocoroides, Coptocatus oblongulus, Coptocatus kinabalu, Tanycricos longiceps, Limnocoris lutzi, Laccocoris hoogstraali, Heleocoris humeralis, Cryphocricos hungerfordi, Ambrysus occidentalis, Pelocoris femoratus, Macrocoris rhantoides, Ilyocoris cimicoides, Naucoris maculatus, Namtokocoris siamensis, Neomacrocoris handlirschi, Paraplea frontalis, Plea minutissima, Helotrephes semiglobosus, Hydrotrephes visayasensis, Hydrotrephes balnearius, Tiphotrephes indicus, *and corixoids species
	(?) Lack of data; Potamocoridae

K30. The number of apical chemosensilla	(0) Four to seven; *Mesovelia furcata *
	(1) Eight to 14 pairs; most species of the Nepomorpha
	(2) More than 15; corixoids species
	(?) Lack of data; Potamocoridae

K31. Types of the labial tip	(0) Smooth; *Mesovelia furcata, *all species of the Nepidae and Belostomatidae, and Nerthrinae (*Nerthra nepaeformis* and *N. macrothorax*) and *Diaprepocoris zealandiae *
	(1) Folded;* Ochterus piliferus, O. marginatus, Gelastocoris oculatus, Aphelocheirus variegatus, A. aestivalis, Anisops camaroonensis, Anisops sardea, Notonecta glauca, Buenoa uhleri, Enithares bergrothi, Nychia sappho, Cheirochela feana, Gestroiella limnocoroides, Coptocatus oblongulus, C. kinabalu, Tanycricos longiceps, Limnocoris lutzi, Laccocoris hoogstraali, Heleocoris humeralis, Cryphocricos hungerfordi, Ambrysus occidentalis, Pelocoris femoratus, Macrocoris rhantoides, Ilyocoris cimicoides, Naucoris maculatus, Namtokocoris siamensis, Neomacrocoris handlirschi, Paraplea frontalis, Plea minutissima, Helotrephes semiglobosus, Hydrotrephes visayasensis, H. balnearius, Tiphotrephes indicus, *and some of the corixoids species
	(?) Lack of data; Potamocoridae

K32. Distribution of mechnosensilla	(0) Less numerous sensilla, grouped and unevenly arranged; *Mesovelia *
	(1) Numerous sensilla, grouped and unevenly arranged; all Belostomatidae
	(2) Densely and evenly arranged sensilla; all Nepidae, Gelastocoridae, and Ochteridae
	(3) Less numerous and numerous, evenly arranged sensilla; all Aphelocheiridae and Naucoridae
	(4) Not numerous and unevenly scattered sensilla; Notonectidae, Pleidae, and Helotrephidae
	(5) Very numerous sensilla arranged in three transverse bands; Diaprepocoridae
	(6) Very numerous sensilla arranged in five transverse bands; Micronectidae
	(7) Very numerous sensilla arranged in six to seven transverse bands; Corixinae and Stenocorixinae
	(8) Numerous sensilla scattered unevenly on the labial surface; Cymatiainae
	(?) Lack of data; Potamocoridae

K33. Shape of the apical ventral plate	(0) Oval shaped; *Mesovelia furcata, *Nepidae, Ochteridae, Aphelocheiridae, Limnocorinae, Helotrephidae, Corixoidea
	(1) Palm shaped; Belostomatidae
	(2) Slim palm shaped; Nerthrinae
	(3) Triangular; Gelastocorinae and Pleidae
	(4) Rectangular; Cheirochelinae, Laccocorinae, Cryphocricinae, and Naucorinae
	(5) Trapezoidal; Notonectidae
	(?) Lack of data; Potamocoridae

K34. Shape of the intercalary sclerites	(0) Large plates (four) overlapping the dorsal and ventral side of the labium; *Mesovelia furcata *
	(1) Two plates placed dorsally, do not reach to the lateral side; Nepidae and Belostomatidae
	(2) Small flaps situated in the middle of the dorsal side; Ochteridae and Gelastocorinae
	(3) Subtriangular shaped, does not overlap the lateral side; Nerthrinae
	(4) Subtriangular shaped, overlaps the lateral side; Aphelocheiridae
	(5) Wide, short flaps with a distinct membrane at the base; Cheirochelinae,
	(6) Wide, short flaps with a slightly distinct membrane at the base; Limnocorinae and Cryphocricinae
	(7) Severely reduced flaps; the membrane is not visible; Laccocorinae, Naucorinae, and Notonectidae
	(8) Lack of intercalary sclerites; Pleidae, Helotrephidae, Potamocoridae, and Corixoidea

K35. Stylet groove of the first segment	(0) Open; *Mesovelia furcata, *Nepidae, Belostomatidae, Aphelocheiridae, Naucoridae, Pleidae, Helotrephidae, and Notonectidae
	(1) Closed; Ochteridae and Gelastocoridae
	(2) Absent (= lack of segment); Corixoidea
	(?) Lack of data; Potamocoridae

K36. Shape of the first segment	(0) Ring shaped, well developed on the dorsal side (medium length, wide); *Mesovelia furcata*, Belostomatidae, Gelastocorinae, Ochteridae, Aphelocheiridae, and Notonectidae
	(1) Ring shaped, weakly developed on the dorsal side (short, narrow); Potamocoridae, Naucoridae, Helotrephidae, and Pleidae
	(2) Reduced on the dorsal side (trace of the segment); Nepidae
	(3) Subtriangular with a deep incision (in); Nerthrinae
	(4) Lack of the segment; Corixoidea

K37. The shape of the second segment, dorsally	(0) The dorsal surface is not divided; *Mesovelia furcata*, Nepidae, Belostomatidae, Nerthrinae, Gelastocorinae, and Ochteridae
	(1) The dorsal surface is divided into a triangular plate, flat; Aphelocheiridae, Potamocoridae, Cheirochelinae, Laccocorinae, Cryphocricinae, Naucorinae*, Buenoa uhleri, Enithares bergrothi, Nychia sappho, Anisops camaroonensis, *and *Anisops sardea *
	(2) The dorsal surface is divided into a triangular plate with a convex plate; *Limnocoris lutzi *
	(3) The dorsal surface is divided into a triangular plate with the nodule; *Notonecta glauca *
	(4) The dorsal surface is divided into a triangular plate with a large nodule; Pleidae and Helotrephidae
	(5) Lack of the segment; Corixoidea

K38. The style groove on the dorsal side of the second segment	(0) Closed along the whole length of the segment; *Mesovelia furcata*, Gelastocoridae, Ochteridae, and Aphelocheiridae
	(1) Without a clear boundary up to the middle of the segment; Nepidae and Belostomatidae
	(2) Open up to the middle of the segment Naucoridae, Notonectidae, Helotrephidae and Pleidae, and Potamocoridae
	(3) Lack of the second segment; Corixoidea

K39. The shape of the second segment, laterally	(0) The lateral surface smooth; *Mesovelia furcata* and the remaining Nepomorpha
	(1) The lateral surface with the winged plate; *Limnocoris lutzi *
	(2) lack of the second segment; Corixoidea

K40. The length of the second segment	(0) Short; *Mesovelia furcata *and the some species of the Nepomorpha
	(1) Long; *Hydrocyrius colombiae, Belostoma bakeri, Belostoma flumineum, Deinostoma dilatatum, *and *Limnogeton fieberi *
	(2) Reduced (or short ventrally); Corixoidea

K41. The length of the third segment	(0) Long; *Mesovelia furcata, *Ochteridae, Aphelocheiridae, and Corixoidea
	(1) Shorter; remaining species of the Nepomorpha

K42. The length of the fourth segment	(0) Short; *Mesovelia furcata* and remaining species of the Nepomorpha
	(1) Long*; *Helotrephidae
	(2) Very short; Corixoidea

K43. The midventral condyle on the I segment	(?) Lack of data; *Mesovelia furcata* and Potamocoridae
	(0) Present; Nepidae, Belostomatidae, Gelastocorinae, Ochteridae, Aphelocheiridae, Naucoridae, Pleidae, Helotrephidae, Notonectidae
	(1) Absent; Nerthrinae and Corixoidea

K44. The midventral condyle on the III segment	(0) Present; *Mesovelia furcata, *Nepidae, Belostomatidae, Cheirochelinae (4 species), and Corixoidea
	(1) Absent; Gelastocoridae, Ochteridae, Aphelocheiridae*, Tanycricos longiceps *(Cheirochelinae), Cryphocricinae, Limnocorinae, Naucorinae, Pleidae, Helotrephidae, and Notonectidae
	(?) Lack of data; Potamocoridae and Laccocorinae

K45. The midventral condyle on the IV segment	(0) Absent; *Mesovelia furcata, *Nepidae, Belostomatidae, Corixoidea, Gelastocoridae, Ochteridae, Aphelocheiridae, and Cheirochelinae
	(1) Present; Cryphocricinae, Limnocorinae, Naucorinae, Pleidae, Helotrephidae, and Notonectidae
	(?) Lack of data; Potamocoridae and Laccocorinae

K46. Dorsal articulation between the second and third segments	(0) Band shaped; *Mesovelia furcata, *Nepidae, Belostomatidae, Gelastocorinae, Aphelocheiridae, Potamocoridae, Naucoridae and Notonectinae, and Potamocoridae
	(1) Distinct condyle present; Ochteridae
	(2) Long and folded membrane; Nerthrinae
	(3) Three cornered; Pleidae and Helotrephidae
	(?) Lack data; Corixoidea

K47. The shape of the labium	(0) Tubular long; *Mesovelia furcata* and most species of the Nepomorpha
	(1) Triangular and short; corixoids species


**Table 3:** The matrix of character states in nepomorphan species and outgroup (Gerromorpha: *Mesovelia furcata*) (0–47).

Name of taxa	Number of characters states 012345678911111111112222222222333333333344444444 01234567890123456789012345678901234567
*Mesovelia furcata *	0000000000000000000000000000000000000000000?0000
*Curicta granulosa *	121128231111110000000001110111102010201001000000
*Borborophyes mayri *	121128231111110000000001110111102010201001000000
*Laccotrephes japonensis *	121128911111110000000001110111102010201001000000
*Nepa cinerea *	121128231111110000000001110111102010201001000000
*Cercotmetus asiaticus *	121122231100001000000001110111102010201001000000
*Ranatra chinensis *	121422231100001000000001110111102010201001000000
*Ranatra linearis *	121422231100001000000001110111102010201001000000
*Belostoma bakeri *	021111111100000110000001010111101110001011000000
*Belostoma flumineum *	021111111100000110000001010111101110001011000000
*Deinostoma dilatatum *	021111111100000110000001010111101110001011000000
*Appasus major *	021111111100000110000001010111101110001011000000
*Hydrocyrius colombiae *	021111151100000110000001010111101110001011000000
*Limnogeton fieberi *	022111111100000010000001010111101110001011000000
*Lethocerus deyrollei *	021115151100000110000001010111101110001001000000
*Ochterus perbosci *	533323001100000110000003000122112021000000001010
*Ochterus marginatus *	533323001100000110000003000122112021000000001010
*Ochterus piliferus *	5?3323001100000110000003000122112021000000001010
*Gelastocoris bufo *	2??123361100000111100003000122112321000001001000
*Gelastocoris oculatus *	213123361100000111100003000122112321000001001000
*Nerthra nepaeformis *	318124062100000110010003000121102231300001011020
*Nerthra macrothorax *	318124062100000110010003000121102231300001011020
*Aphelocheirus variegatus *	547224481100000110001003000122113040010000001000
*Aphelocheirus aestivalis *	547224481100000110001003000122113040010000001000
*Cheirochela feana *	644124981100000010000003012122113450112001000000
*Gestroiella limnocoroides *	644124981000000010000003012122113450112001000000
*Coptocatus oblongulus *	?45121381100000010000003011122113450112001000000
*Coptocatus kinabalu *	?45121381100000010000003011122113450112001000000
*Tanycricos longiceps *	7?4121751100000010000003011122113450112001001000
*Laccocoris hoogstraali *	75412578110000001000000301212211347011200100??00
*Heleocoris humeralis *	75412578110000001000000301212211347011200100??00
*Limnocoris lutzi *	754123783100000000000103011122113460122101001100
*Cryphocricos hungerfordi *	754125781100000000000103013122113460112001001100
*Ambrysus occidentalis *	754125641000000000000103012122113460112001001100
*Ilyocoris cimicoides *	654126781100000000000103012122113470112001001100
*Pelocoris femoratus *	755126781100000000000103012122113470112001001100
*Macrocoris rhantoides *	754125781100000000000103012122113470112001001100
*Naucoris maculatus *	?54125781100000000000103023122113470112001001100
*Neomacrocoris handlirschi *	76612578?100000000000103013122113470112001001100
*Namtokocoris siamensis *	?54128711100000000000103013122113470112001001100
*Plea minutissima *	859126884000000000000002020122114380142001001130
*Paraplea frontalis *	859126884000000000000002020122114380142001001130
*Helotrephes semiglobosus *	959128541000000000000001020122114080142001101130
*Hydrotrephes visayasensis *	959122571000000000000001010122114080142001101130
*Hydrotrephes balnearius *	959126881000000000000001010122114080142001101130
*Tiphotrephes indicus *	959126881000000000000000000122114080142001101130
*Anisops camaroonensis *	758127911100000000000001010122114570012001001100
*Anisops sardea *	758127911100000000000001010122114570012001001100
*Buenoa uhleri *	758127581100000000000001020122114570012001001100
*Notonecta glauca *	758127421100000000000002010122114570032001001100
*Enithares bergrothi *	758127411100000000000001020122114570012001001100
*Nychia sappho *	75812798?100000000000001010122114570012001001100
*Agraptocorixa hyalinipennis *	4794238651000000100000130301022170824532212100?1
*Corixa punctata *	4794238651000000100000130301022170824532212100?1
*Corixa affinis *	4794238651000000100000130301022170824532212100?1
*Ectemnostegella montana *	4794238651000000100000130301022170824532212100?1
*Hesperocorixa linnaei *	4794238651000000100000130301022170824532212100?1
*Sigara lateralis *	4794238651000000100000130301022170824532212100?1
*Cymatia coleoptrata *	4794238651000000100000130301022180824532212100?1
*Stenocorixa protrusa *	4794238651000000100000130301022170824532212100?1
*Diaprepocoris zealandiae *	47942386510000001000001303010220508245322?2100?1
*Micronecta quadristrigata *	4794238651000000100000130301022160824532212100?1
*Potamocoridae *	?49????????????????????????1??????8?1120010???00;

Symbols: (?) unknown data.

## References

[1] Štys P, Kerzhner IM (1975). The rank and nomenclature of higher taxa in recent Heteroptera. *Acta Entomologica Bohemoslovaca*.

[2] Slater JM, Parker S (1982). Hemiptera. *Synopsis and Classification of Living Organism*.

[3] Schuh RT, Slater JA (1995). True bugs of the world (Hemiptera: Heteroptera). *Classification and Natural History*.

[4] Sweet MH (2006). Justification for the Aradimorpha as an infraorder of the suborder Heteroptera (Hemiptera, Prosorrhyncha) with special reference to the pregenital abdominal structure. *Denisia 19, Zugleich Kataloge der OÖ*.

[5] Sweet MH, Schaefer CW (1996). Comparative external anatomy of the pregenital abdomen of the Hemiptera. *Studies on Hemipteran Phylogeny*.

[6] Schuh RT (1979). Evolutionary Trends in Heteroptera—Part II: Mouthpart-structures and Feeding Strategies. *Systematic Zoology*.

[7] Štys P (1985). The present state of the beta-taxonomy in Heteroptera. *Práce Slovenská Entomologická Spolocnostśva, Bratislva*.

[8] Štys P (1989). Phylogenetic systematics of the most primitive true bugs (Heteroptera: Enicocephalomorpha, Dipsocoromorpha). *Práce Slovenská Entomologická Spolocnostśva, Bratislva*.

[9] Zrzavy J (1992). Evolution of antennae and historical ecology of the hemipteran insects (Paraneoptera). *Acta Entomologica Bohemoslovaca*.

[10] Mahner M (1993). *Systema Cryptoceratorum Phylogeneticum (Insecta, Heteroptera)*.

[11] Scherbakov DE, Popov YA, Rasnitsyn AP, Quicke DLJ (2002). Superorder cimicidea laicharting, 1781. Order Hemiptera linne, 1758. The bugs, cicadas, plantlice, scale insects, etc. *History of Insects*.

[12] Wheeler WC, Schuh RT, Bang R (1993). Cladistic relationships among higher groups of Heteroptera: congruence between morphological and molecular data sets. *Entomologica Scandinavica*.

[13] Yang C-T (2002). Preliminary thoughts on the phylogeny of Coleorrhyncha—Heteroptera (Hemiptera). *Formosan Entomologist*.

[14] Xie Q, Tian Y, Zheng L, Bu W (2008). 18S rRNA hyper-elongation and the phylogeny of Euhemiptera (Insecta: Hemiptera). *Molecular Phylogenetics and Evolution*.

[15] Damgaard J (2008). Phylogeny of the semiaquatic bugs (Hemiptera-Heteroptera, Gerromorpha). *Insect Systematics & Evolution*.

[16] Li M, Tian Y, Zhao Y, Bu W (2012). Higher level phylogeny and the first divergence time estimation of Heteroptera (Insecta: Hemiptera) based on multiple genes. *PLoS ONE*.

[17] Andersen NM (1982). *The Semiaquatic Bugs (Hemiptera, Gerromorpha): Phylogeny, Adaptations, Biogeography and Classification*.

[18] van Doesburg PH (1984). A new species of *Potamocoris* Hungerford, 1941 from suriname (Heteroptera: Naucoridae). *Zoologische Mededelingen*.

[19] Cobben RH (1978). *Evolutionary Trends in Heteroptera—Part 2: Mouthpart-Structures and Feeding Strategies*.

[20] Esaki T, China WE (1927). A new family of aquatic Heteroptera. *Transactions of the Royal Entomological Society of London*.

[21] China WE (1955). The evolution of the water bugs. Symposium on organic evolution. *Bulletin of the National Institute of Science in India*.

[22] Parsons MC (1966). Labial skeleton and musculature of the Hydrocorisae (Heteroptera). *Canadian Journal of Zoology*.

[23] Parsons MC (1969). The labium of *Aphelocheirus aestivalis* F. as compared with that of typical Naucoridae (Heteroptera). *Canadian Journal of Zoology*.

[24] Cobben RH (1968). *Evolutionary Trends in Heteroptera—Part I: Eggs, Architecture of the Shell, Gross Embrylology, and Eclosion*.

[25] Popov YA (1971). Historical development of the hemipterous infraorder Nepomorpha. *Trudy Paleontological Institute Academy of Science, Nauk, USSR*.

[26] Rieger C (1976). Skeleton and musculature of the head and prothorax of *Ochterus marginatus* Latreille—contribution towards clarification of the phylogenetic relationships of the Ochteridae (Insecta, Heteroptera). *Zoomorphologie*.

[27] Hebsgaard MB, Andersen NM, Damgaard J (2004). Phylogeny of the true water bugs (Nepomorpha: Hemiptera-Heteroptera) based on 16S and 28S rDNA and morphology. *Systematic Entomology*.

[28] Hua J, Li M, Dong P, Cui Y, Xie Q, Bu W (2009). Phylogenetic analysis of the true water bugs (Insecta: Hemiptera: Heteroptera: Nepomorpha): evidence from mitochondrial genomes. *BMC Evolutionary Biology*.

[29] Li M, Wang J, Tian X, Xie Q, Liu H, Bu W (2012). Phylogeny of the true water bugs (Hemiptera-Heteroptera: Nepomorpha) based on four Hox genes. *Entotaxonomia*.

[30] Polhemus JT, Aukema B, Rieger C (1995). Enicocephalomorpha, Dipsocoromorpha, Gerromorpha and Leptopodomorpha. *Catalogue of the Heteroptera of the Palaearctic Region*.

[31] Štys P, Jansson A (1988). Check-list of recent family-group and genus-group names of Nepomorpha (Heteroptera) of the world. *Acta Entomologica Fennica*.

[32] Nieser N (2002). Guide to aquatic Heteroptera of Singapore and Peninsular Malaysia. IV. Corixoidea. *The Raffles Bulletin of Zoology*.

[33] Chen PP, Nieser N, Zettel H (2005). *The Aquatic and Semiaquatic Bugs (Heteroptera: Nepomorpha & Gerromorpha) of Malesia*.

[34] Fent M, Kment P, Çamur-Elipek B, Kirgiz T (2011). Annotated catalogue of Enicocephalomorpha, Dipsocoromorpha, Nepomorpha, Gerromorpha, and Leptopodomorpha (Hemiptera: Heteroptera) of Turkey, with new records. *Zootaxa*.

[35] Nieser N, Chen PP, Rabitsch W Two new genera and a new subfamily of Micronectidae (Heteroptera, Nepomorpha) from Brazil. *Hug the Bug—for Love of True Bugs*.

[36] Tinerella PP (2008). Taxonomic revision and systematics of New Guinea and Oceania pygmy water boatmen (Hemiptera: Heteroptera: Corixoidea: Micronectidae). *Zootaxa*.

[37] Weirauch C, Schuh RT (2011). Systematics and evolution of Heteroptera: 25 years of progress. *Annual Review of Entomology*.

[38] Heckman CW, Heckman CW (2011). *Encyclopedia of South American Aquatic Insects: Hemiptera—Heteroptera: Illustrated Keys to Known Families, Genera, and Species in South America*.

[39] Kment P, Jindra Z, Grozneva S, Simov N (2008). Review of the family Gelasotocoridae (Heteroptera: Nepomorpha) of South-Eastern Asia. *Advances in Heteroptera Research*.

[40] Pendergrast JG (1957). Studies on the reproductive organs of Heteroptera with a consideration of their bearing on classification. *Transactions of the Royal Entomological Society of London*.

[41] Polhemus DA, Polhemus JT, Sites R (2008). A revision of the Indochinese genera *Cheirochela* and *Gestroiella* (Heteroptera: Naucoridae), and a review of the tribe Cheirochelini. *The Raffles Bulletin of Zoology*.

[42] Reuter OM (1912). *Bemerkungen über Mein Neues Heteropteren System*.

[43] Sutton MF (1947). Feeding mechanism of water-bugs. *Nature*.

[44] Sutton MF (1951). On the food, feeding mechanism and alimentary canal of Corixidae (Hemiptera, Heteroptera). *Proceedings of the Zoological Society of London*.

[45] Papáček M (2001). Small aquatic and ripicolous bugs (Heteroptera: Nepomorpha) as predators and prey: the question of economic importance. *European Journal of Entomology*.

[46] Reuter OM (1910). *Neue Beiträge zur Phylogenie und Systematik der Miriden Nebst Einleitenden Bemerkungen über die Phylogenie der Heteropteren-Familien*.

[47] Menke AS (1979). The semiaquatic and aquatic Hemiptera of California. *Bulletin of the California Insect Survey*.

[48] Parsons MC (1966). Modifications of the food pumps of Hydrocorisae (Heteroptera). *Canadian Journal of Zoology*.

[49] Jaczewski T (1924). *Revision of the Polish Corixidae*.

[50] Stonedahl GM, Lattin JD (1986). *The Corixidae of Oregon and Washington (Hemiptera: Heteroptera)*.

[51] Wollmann K (2000). Corixidae (Hemiptera, Heteroptera) in acidic mining lakes with pH ≤ 3 in Lusatia, Germany. *Hydrobiologia*.

[52] Popham EJ (1960). On the respiration of aquatic Hemiptera: Heteroptera with special reference to the Corixidae. *Proceedings of the Zoological Society of London*.

[53] Cassis G, Silveira R (2001). A revision and phylogenetic analysis of the *Nerthra alaticollis* species-group (Heteroptera: Gelastocoridae: Nerthrinae). *Journal of the New York Entomological Society*.

[54] Griffith ME (1945). The environment, life history, and structure of the water boatman, *Ramphocorixa acuminata* (Uhler) (Hemiptera, Corixidae). *The University of Kansas Science Bulletin*.

[55] Benwitz G (1956). Der kopf von corixa punctata ILL., (geoffroyi Leach) (Hemiptera-Heteroptera). *Zoologische Jahrbücher Abteilung für Anatomie und Ontogenie der Tiere*.

[56] Puchkova LV (1963). Labium of aquatic Hemipter-Heteroptera. *Dopovidi Akademiï nauk Ukraïn'skoï RSR*.

[57] Brożek J (2013). A comparison of the external and internal maxilla and mandible morphology of water bugs (Hemiptera: Heteroptera: Nepomorpha). *Zootaxa*.

[58] Brożek J (2013). Comparative analysis and systematic mapping of the labial sensilla in the Nepomorpha (Heteroptera: Insecta). *The Scientific World Journal*.

[59] Brożek J (2013). Deliberations on the external morphology and modification of the labial segments in the Nepomorpha (Heteroptera: Insecta) with notes on the phylogenetic characteristics. *The Scientific World Journal*.

[60] Goloboff PA NONA, version 2.0.

[61] Nixon KC WinClada (program).

[62] Goloboff PA (1993). Estimating character weights during tree search. *Cladistics*.

[63] Goloboff PA (1997). Self-weighted optimization: tree searches and character state reconstructions under implied transformation costs. *Cladistics*.

[64] Swofford DL (1998). *PAUP 4.0: Phylogenetic Analysis Using Parsimony (and Other Methods)*.

[65] Goloboff PA PeeWee, version 2.6. Parsimony and implied weights.

[66] Bremer K (1994). Branch support and tree stability. *Cladistics*.

[67] Goloboff PA, Farris J, Nixon K T.N.T. tree analysis using new technology. http://www.zmuc.dk/public/phylogeny/tnt.

[68] Felsenstein J (1985). Confidence limits on phylogenies: an approach using the bootstrap. *Evolution*.

[69] Altner H, Prillinger L (1980). Ultrastructure of invertebrate chemo-, thermo-, and hygroreceptors and its functional significance. *International Review of Cytology*.

[70] Chapman RF, Chapman RF (1998). Mechanoreception chemoreception. *The Insects: Structure and Function*.

[71] Shields VDC, Méndez-Vilas A, Díaz J (2010). High resolution ultrastructural investigation of insect sensory organs using field emission 
scanning electron microscopy. *Microscopy: Science, Technology, Applications and Education*.

[72] Zacharuk RY (1980). Ultrastructure and function of insect chemosensilla. *Annual Review of Entomology*.

[73] Hungerford HB (1941). A remarkable new naucorid water bug (Hemiptera). *Annals of the Entomological Society of America*.

[74] Hungerford HB (1942). *Coleopterocoris*, an interesting new genus of the subfamily Potamocorinae. *Annals of the Entomological Society of America*.

[75] la Rivers I (1950). A new species of the genus *Potamocoris* from Honduras. *Proceedings of the Entomological Society of Washington*.

[76] la Rivers I (1969). *New Naucorid Taxa*.

[77] Parsons MC (1959). *Skeleton and Musculature of the Head of Gelastocoris Oculatus (Fabricius) (Hemiptera-Heteroptera)*.

[78] Börner C (1904). Zur systematik der hexapoden. *Zoologischer Anzeiger*.

[79] Quadri MAH (1951). On the anatomy of the mouth-parts and the mode of feeding in the aquatic bugs (Cryptocerata). *Proceedings of the Zoological Society*.

[80] Cassis G, Gross GF (1995). *Hemiptera: Heteroptera (Coleorrhyncha to Cimicomorpha)*.

